# Bioactivities and Mechanisms of Action of Sinomenine and Its Derivatives: A Comprehensive Review

**DOI:** 10.3390/molecules29020540

**Published:** 2024-01-22

**Authors:** Wen Hou, Lejun Huang, Hao Huang, Shenglan Liu, Wei Dai, Jianhong Tang, Xiangzhao Chen, Xiaolu Lu, Qisheng Zheng, Zhinuo Zhou, Ziyun Zhang, Jinxia Lan

**Affiliations:** 1 College of Pharmacy, Gannan Medical University, Ganzhou 341000, China; twenhou@gmu.edu.cn (W.H.); hhuang@gmu.edu.cn (H.H.); liushl5@gmu.edu.cn (S.L.); daiwei@gmu.edu.cn (W.D.); chenxz20@gmu.edu.cn (X.C.); luxiaolu@gmu.edu.cn (X.L.); qishengzheng@edu.gmu.com (Q.Z.); zhou3110734209@163.com (Z.Z.); 19324828384@163.com (Z.Z.); 2 College of Rehabilitation, Gannan Medical University, Ganzhou 341000, China; 18379811337@163.com; 3 Laboratory Animal Engineering Research Center of Ganzhou, Gannan Medical University, Ganzhou 341000, China; tangjianhong@gmu.edu.cn; 4 College of Public Health and Health Management, Gannan Medical University, Ganzhou 341000, China

**Keywords:** sinomenine, derivatives, antitumor, anti-inflammatory, immunosuppressive, neuroprotection

## Abstract

Sinomenine, an isoquinoline alkaloid extracted from the roots and stems of *Sinomenium acutum*, has been extensively studied for its derivatives as bioactive agents. This review concentrates on the research advancements in the biological activities and action mechanisms of sinomenine-related compounds until November 2023. The findings indicate a broad spectrum of pharmacological effects, including antitumor, anti-inflammation, neuroprotection, and immunosuppressive properties. These compounds are notably effective against breast, lung, liver, and prostate cancers, exhibiting IC_50_ values of approximately 121.4 nM against PC-3 and DU-145 cells, primarily through the PI3K/Akt/mTOR, NF-κB, MAPK, and JAK/STAT signaling pathways. Additionally, they manifest anti-inflammatory and analgesic effects predominantly via the NF-κB, MAPK, and Nrf2 signaling pathways. Utilized in treating rheumatic arthritis, these alkaloids also play a significant role in cardiovascular and cerebrovascular protection, as well as organ protection through the NF-κB, Nrf2, MAPK, and PI3K/Akt/mTOR signaling pathways. This review concludes with perspectives and insights on this topic, highlighting the potential of sinomenine-related compounds in clinical applications and the development of medications derived from natural products.

## 1. Introduction

**Figure 1:**
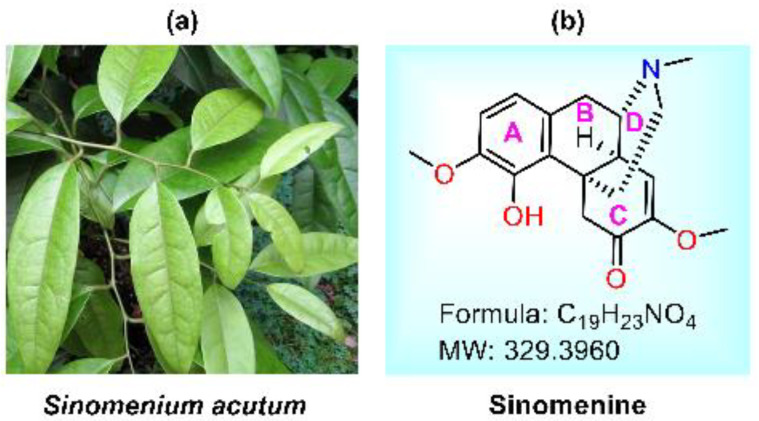
(**a**) Picture of *sinomenium acutum*; (**b**) the structure of sinomenine.

Natural products (NPs) serve as a crucial foundation for contemporary pharmaceuticals, with approximately 65% of clinical drugs being derived either directly or indirectly from them [1,2,3]. Despite their significance, NPs often present challenges, such as low activity or ambiguous mechanisms of action [4]. Consequently, modifying NPs to enhance activities and elucidate targets or mechanisms of action is of paramount importance [5,6]. Sinomenine (SIN, **1**), an isoquinoline alkaloid, is isolated from the roots and stems of *Sinomenium acutum* or *Caulis Sinomenii*. Its chemical name is (9α, 13α, 14α)-7,8-dihydro-4-hydroxy-3,7-dimethoxy-17-methylmorpholinan-6-one. The compound’s structure comprises four rings (A, B, C, and D rings): the A ring is a benzene ring, the B ring is a semi-chair six-membered ring linked to the A ring, the C ring is a twisted chair six-membered ring with an α, β-unsaturated ketone structure connected to the B ring, and the D ring is a nitrogen-containing chair six-membered ring positioned below the B ring. Its molecular formula is C_19_H_23_NO_4_ (Figure 1b) [7]. Studies have shown that sinomenine is blood–brain barrier (BBB)-permeable [8,9].

**Figure 2:**
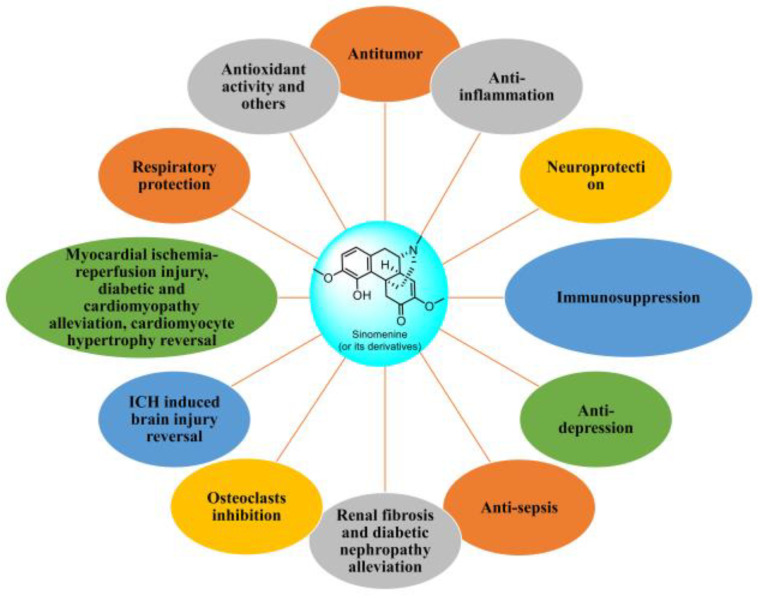
Bioactivities of sinomenine or its derivatives.

Most derivatives of sinomenine result from structural modifications of their A, B, C, and D rings. Sinomenine exhibits low water solubility [10], leading to the development of sinomenine hydrochloride (SH) to enhance its water solubility and drug-like properties. Numerous biological studies have confirmed a wide array of biological activities for these compounds, including antitumor [11], anti-inflammatory [12], analgesic [13], immunomodulatory [14], neuroprotective [15], and cardioprotective effects [16], as summarized in Figure 2. These properties render them potential candidates for clinical medications. Although a recent online publication reviewed the pharmacological activity of sinomenine [17], a systematic review focusing on combination strategies and the development of sinomenine derivatives remains crucial. Thus, this article reviews the research progress in the bioactivities and action mechanisms of sinomenine and its derivatives, aiming to enlighten the clinical application of sinomenine-related compounds. It has been demonstrated that they possess multi-pharmacological effects such as antitumor, anti-inflammation, neuroprotection, and immunosuppressive properties. Additionally, they exhibit anti-inflammatory and analgesic effects and are utilized in treating rheumatic arthritis. Moreover, they play a significant role in cardiovascular and cerebrovascular protection, as well as organ protection.

## 2. Methodology

### 2.1. Inclusion Criteria

An extensive survey of the “Sinomenine”, “derivatives”, “antitumor”, “anti-inflammatory”, “immunosuppressive”, “neuroprotection”, “bioactivity”, etc., was conducted in scientific databases, including Web of Science, PubMed, Science Direct, and Google Scholar. The search terms “Sinomenine”, “derivatives”, “bioactivity”, etc., were used for data collection. In total, 230 publications were included from 1985 to November 2023. From those studies on the bioactivity and/or mechanism of action of sinomenine and/or its derivatives, some articles were removed.

### 2.2. Exclusion Criteria

Articles only related to the synthesis or isolation of sinomenine and/or its derivatives without activity assessment were excluded.

## 3. Antitumor Activity

### 3.1. Antitumor Activity of SIN When Used Alone

**Figure 3:**
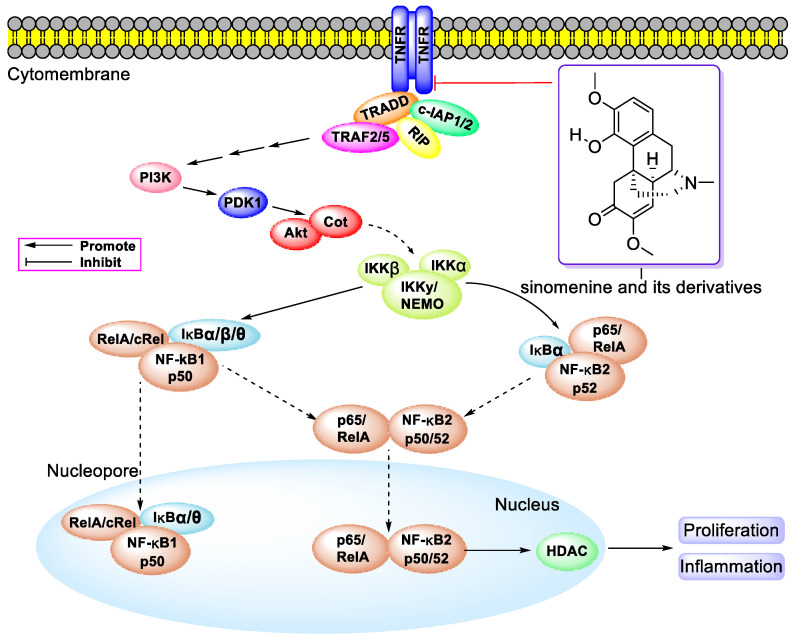
NF-κB pathway related to the antitumor and anti-inflammation activities of sinomenine and its derivatives.

Cancer ranks as the second leading cause of mortality worldwide. Consequently, the discovery of effective antitumor agents holds significant importance [18]. In 2006, X.J. Li et al. discovered that SIN (0.125–1 mM) curtailed the proliferation of IL-1β-activated human synovial sarcoma cells (Hs701.T) and attenuated the expression of *IL-6*, *PLGF*, *Daxx*, and *HSP27*, which are implicated in cell proliferation, angiogenesis, and vascular remodeling [19]. In 2009, Y. Q. Ou et al. delineated that SIN (0.01–1.00 mM) suppressed the invasion and migration of activated human monocyte THP-1 (A-THP-1) cells by diminishing CD147 and MMP-2/-9 expression [20]. The following year, T.S. Jiang and associates reported that SIN (242.9–607.2 μM) inhibited the proliferation of NCI-H460 cells and instigated mitochondrial-mediated apoptosis through the activation of caspase-3/-9, increased Bax levels, decreased BcL-2 levels, and thus elevated the Bax/BcL-2 ratio, leading to cellular apoptosis [21]. In 2011, J. Fan and team demonstrated that SIN (0.006–0.304 μM) induced apoptosis in prostate cancer cells PC-3 and DU-145 by downregulating prostaglandin E2 (PGE2), cyclooxygenase 2 (COX-2), and NF-κB (as shown in Figure 3) and reducing NF-κB-p65 levels (IC_50_ value was 121.4 μM) [22]. In the subsequent year, L.P. Zhou’s group observed that SIN (607.1 μM) inhibited NCI-H460 cell proliferation by obstructing the AKT and ERK1/2 pathways [23].

**Figure 4:**
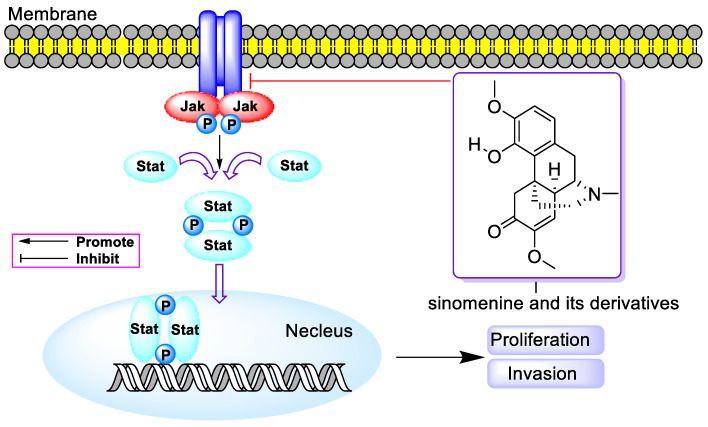
JAK/STAT pathway related to the anti-proliferation and anti-invasion activities of sinomenine and its derivatives.

In 2015, L.Q. Song et al. revealed that SIN (0.25–1 mM) impeded the binding of NF-κB to IκB and the nuclear translocation of NF-κB in MDA-MB-231 cells, as depicted in Figure 3. It also downregulated mesenchymal markers such as vimentin and tenogenic protein-C, along with cytokines like cholecystokinin (CCK), monocyte chemotactic protein-1 (MCP-1), and IL-11. This inhibition was achieved by decreasing IL-4/miR-324–5p and upregulating CUEDC2, resulting in suppressed proliferation, invasion, and migration of breast cancer cells (MDA-MB-231 and 4T1) [24]. Following this, S.L. Jiang et al. reported that SIN (0.125–2.0 mM) reduced viability and induced apoptosis in A549 cells by diminishing JAK2, STAT3, p-STAT3, Snail, N-cadherin, and vimentin while increasing E-cadherin, highlighting the crucial role of the STAT3 signaling pathway in SIN’s anti-proliferative and anti-invasive activities, as illustrated in Figure 4 [25]. In the same year, T. Xie’s group discovered that SIN (20–400 µM) curbed tumor invasion and metastasis by inhibiting CXCR4 and STAT3 phosphorylation and downregulating MMP-2/-9, VEGF, RANKL, and CD147, thereby impeding the invasion and metastasis of osteosarcoma and hindering angiogenesis in human umbilical vein endothelial cells (HUVEC). At a dosage of 150 mg/kg, SIN hindered osteosarcoma metastasis by inhibiting RANKL-mediated osteolysis, regulating intracellular alkaline phosphatase (ALP) expression, improving cortical bone integrity, and reducing osteoclast numbers [26]. The following year, Y.M. Jiang et al. demonstrated that SIN (0.125–0.5 mM) reduced cell viability in U87 and SF767 cells through reactive oxygen species (ROS) induction, Akt/mTOR pathway suppression, and JNK pathway activation, thereby inducing autophagy. Furthermore, SIN facilitated the nuclear translocation of transcription factor EB (TFEB) and enhanced lysosome production. At dosages of 75 or 150 mg/kg, it increased cathepsin B and D levels and reduced p62 in tumor tissues without causing behavioral or morphological abnormalities in vital organs such as the lungs, liver, pancreas, and kidneys [27].

**Figure 5:**
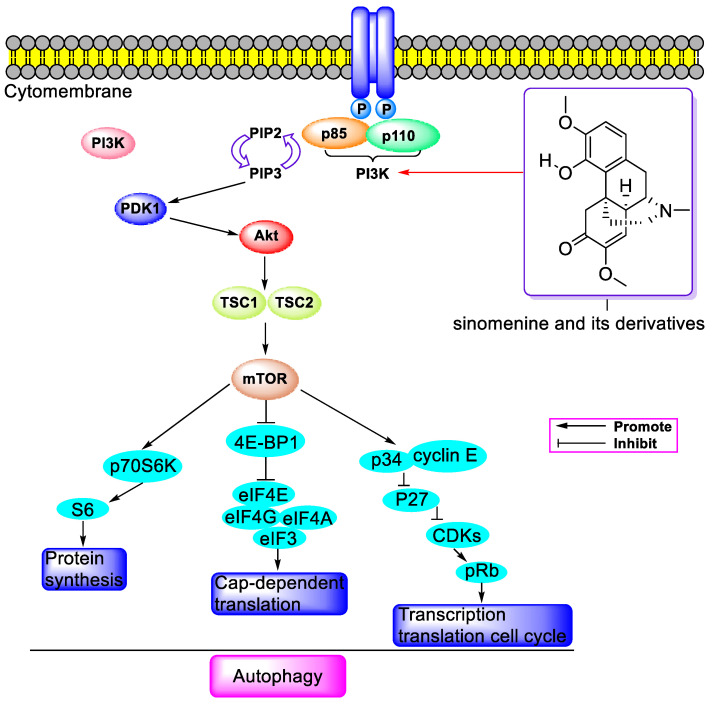
PI3K/AKT/mTOR pathway related to autophagy induction activity of sinomenine and its derivatives.

In 2018, Z. Sun et al. reported that SIN (0–100 μM) decreased the viability of B melanoma 16-F10 cells by increasing the Bax/BcL-2 ratio, Beclin 1, and the eLC3II/LC3I ratio, reducing p-p62/SQSTML, and enhancing the PI3K/Akt/mTOR-dependent autophagy pathway (as shown in Figure 5), thereby promoting cell apoptosis. SIN (100 mg/kg) was also effective in melanoma xenograft mice [28]. In the same year, studies found that SIN (16 mM) inhibited the proliferation of glioma cells (U87 and U251) and induced G0/G1 cell cycle arrest and apoptosis by elevating p53 and decreasing SIRT1. SIN (100 mg/kg) impeded the growth of U87 xenograft tumors. However, lower-to-moderate concentrations (up to 32 mmol/L) of sinomenine did not exhibit significant cytotoxicity to normal astrocytes. Sinomenine significantly inhibited the growth of colon cancer xenografts in nude mice without causing notable side effects [29]. SIN (0.5 mM) curbed the secretion of MMP-2 and vimentin and reduced IL-11 by inhibiting the activation of the NF-κB and NF-κB-mediated Sonic hedgehog signaling pathway, leading to the inhibition of the proliferation and migration of MDA-MB-231 cells. SIN (15 mg/kg) also hindered the progression of lung metastasis in breast cancer. SIN was proven to be more effective than cyclopamine (10 mM in vitro and 120 mg/kg in vivo) in curbing the lung metastasis of breast cancer in vivo and in vitro and in inhibiting NF-κB activation and the NF-κB-mediated Shh signaling pathway [30]. In 2018, G.L. Gao et al. found that SIN (4 μM) reduced PCNA, CyclinD1, and CDK4, increased p16, and increased cleaved-caspase-3/-9 in MDA-MB-231 and MCF-7 cells by elevating miR-29, leading to the downregulation of p-JNK, p-MEK, and p-ERK, ultimately inhibiting the proliferation, migration, and invasion of breast cancer cells [31]. In the same year, H.F. Yuan et al. reported that SIN blocked AMPK and Wnt/β-catenin of MMP-9 by elevating miR-204 [32]. In the following year, it was reported that SIN (25–100 µM) inhibited cell migration and decreased p-Histone H3 (Ser10), thereby impeding the growth of non-small-cell lung cancer (NSCLC) cells by inhibiting the activity of Akt and downstream kinase S6, reducing hexokinases 2 (HK2), and hindering glycolysis. Additionally, SIN (40 mg/kg) lessened tumor volume and weight in mice transplanted with HCC827 and H1975 tumor cells [33].

Two years prior, W. Yang et al. demonstrated that SIN (2 and 4 mM) inhibited AMPK and STAT3 phosphorylation by downregulating cell-membrane-associated ring finger protein 1 (MARCH1) and blocked the hepatocellular carcinoma cell (Hep3B and HepG2 cells) cycle in the G0/G1 phase, leading to the inhibition of cell proliferation and induction of apoptosis [34]. Mechanism investigations revealed that SIN (0.25–1 mM) also curbed the invasion of hypoxia-induced breast cancer SP cells (MDA-MB-231 and MCF-7) by reducing N-cadherin vimentin, and MMP-2/-9 and inhibiting the phosphorylation of PI3K, Akt, and mTOR [35]. X.Y. Qu and team reported that SIN inhibited the proliferation (IC_50_ = 1.56 mM) and colony-formation ability of ovarian cancer HeyA8 cells by downregulating CDK1, reducing p-CDK (Thr161) and p-Histone H3 (Ser10) [36]. The previous year, F. Gao et al. reported the antitumor activity and mechanism of Mufangji decoction (MFJD), of which SIN is a component in preventing lung cancer. The results showed that SIN (3 μM) enhanced the phagocytosis of neutrophils and increased MPO and ROS to promote neutrophil-related immune surveillance [37]. Other studies revealed that SIN inhibited the proliferation of SK-Hep1 and induced apoptosis, decreasing PCNA, PI3K p85α, AKT1, BcL-2, and pro-caspase-3/-9 and increasing cleaved caspase-9/-3 in tumor tissue. They found that the PI3K/AKT1 pathway mediated mitochondrial-related apoptosis in HCC [38]. In the same year, D.Z. Duan and associates reported that SIN (0.25–1 mM) reduced the activity of HeLa cells, increased the activity of caspase-3, and induced apoptosis. Additionally, administering SIN (70 and 140 mg/kg) inhibited the activity of thioredoxin reductase (TrxR), enhanced ROS production, and induced apoptosis, thus impeding cervical tumor growth in vivo [39]. In the same year, L.Q. Song et al. announced the therapeutic effect of SIN on breast cancer, finding that SIN (0.75 mM) hindered hypoxia-induced vasculogenic mimicry (VM) formation and metastasis of breast cancer SP cells by suppressing the hypoxia-induced downregulation of miR-340–5P and activation of the SIAH2/HIF-1α pathway and inhibiting the epithelial-to-mesenchymal transition of breast cancer SP cells [40]. J. Zhang’s group reported that SIN (120 mg/kg) inhibited azoxymethane (AOM)/dextran sulfate sodium (DSS)-induced rectal neoplasia in CAC mice. It lowered pro-inflammatory cytokine levels of IL-1β and TNF-α and enhanced carnitine palmitoyl transferase 1A (CPT1A) and lysophosphatidylcholine acyltransferase 3 (LPCAT3), thereby attenuating the anti-proliferative effect of SIN (2.5 mM) on human colon cancer cells (HT-29, HCT-116, and SW-480) [41]. The comprehensive aspects of sinomenine’s antitumor activity are summarized in Table 1.

### 3.2. Antitumor Activity of Sinomenine Hydrochloride

**Figure 6:**
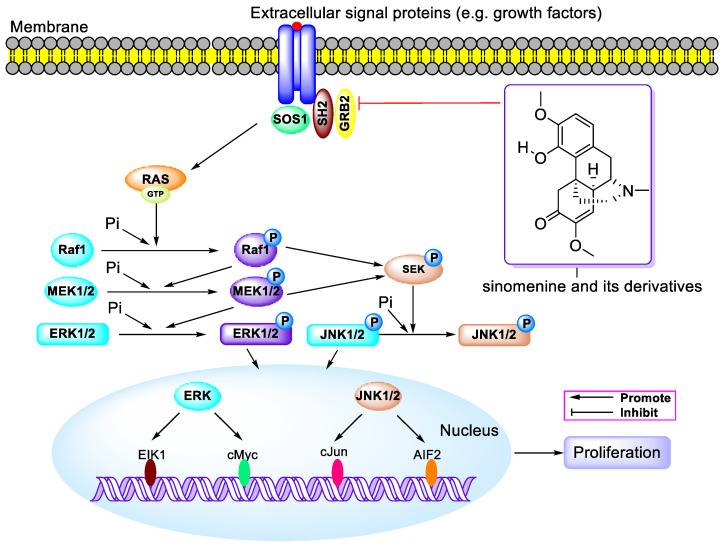
MAKP pathway related to the antitumor activity of sinomenine (or SH) and its derivatives.

The hydrochloride form of alkaloids is a common strategy used to enhance their solubility in water. X.L. Lu et al. synthesized sinomenine hydrochloride (SH) and discovered that SH (0.5–2 µM) inhibited the growth of Hep3B and SMMC7721 cells, leading to G1 phase arrest through the augmentation of p21. It also increased the Omi/HtrA2 ratio, reduced the BcL-2/Bax ratio, activated the caspase cascade and PARP, and decreased survivin protein. Furthermore, SH at doses of 50, 100, and 150 mg/kg demonstrated antitumor activity without significant toxic side effects [42]. A year later, X. Li and team reported that SH exhibited anti-tumor effects against MDA-MB-231 and MCF-7 cells with IC_50_ values of 1.33 mM and 1.51 mM, respectively. It induced G0/G1 phase arrest, decreased cyclin D1, cyclin E, CDK4, MCM7, and p-Rb, and increased p21 and p27. SH also induced DNA damage in breast cancer cells through the ATM/ATR-Chk1/Chk2 and MAPK pathways (as shown in Figure 6), elevated p-ERK, p-JNK, and p-P38, and increased ROS levels. In vivo, SH at 75 and 150 mg/kg also showed antitumor activity in nude mice [43].

In 2017, B. Zhao and co-workers demonstrated that SH (20 and 80 µM) inhibited the growth of clear-cell renal cell carcinoma (ccRCC) cells (ACHN and 786-O) with IC_50_ values of 76.8 μM and 85.5 μM, respectively, for ACHN and 786-O cells. SH reduced MMP-2/-9 levels and inhibited ccRCC cell migration, invasion, and angiogenesis. It also reduced Snail1 and Twist expression and blocked the epithelial–mesenchymal transition (EMT) and EMT-related transcription factors in ccRCC cells by targeting Smad [44]. In 2018, Y.M. Jiang and team found that SH mitigated the metastasis of U87 and SF767 cells both in vitro (0.25 mM) and in vivo (75 mg/kg) by inhibiting NF-κB activation, decreasing MMP-2/-9, triggering endoplasmic reticulum (ER) stress and autophagy, and reversing endogenous and exogenous EMT [45]. In the same year, W. Zhang et al. reported the benefits of SH polylactic acid microcapsules, which inhibited both bacteria and MDA-MB-231 cells. It (15.2 mM) exhibited a sustained release effect, a slower release rate, good biocompatibility, and no apparent cytotoxicity to normal L929 cells [46]. Concurrently, D. Zhang et al. announced the radiosensitive effect of SH on cervical cancer cells. They found that SH (1 mM) accumulated DNA damage and modulated the double-strand break repair pathway by inhibiting the DNA damage response (DDR) factors KU80 and RAD51. It (100 mg/kg) enhanced the IR-induced growth inhibition of cervical graft tumors, and the combination of SH with IR treatment significantly reduced tumor growth compared with IR alone [47]. In the same year, S.B. Fu and team discovered that SH inhibited the growth of esophageal squamous cell carcinoma (ESCC) cells (IC_20_ values were 0.3 mM and 0.4 mM for Eca109 and EC970), induced G2/M phase arrest, downregulated the BcL-2/Bax ratio, cyclin B1, CDK1, Ku86/70, and Rad5 to enhance radiosensitivity. SH (75 mg/kg) combined with radiotherapy significantly increased apoptosis in ESCC cells [48].

C.H. Shen’s group found that SH (0.25 mM) inhibited the survival and invasion of hepatocellular carcinoma SK-Hep1 via the ERK1/2/MMP2/9 pathway [49]. J. Zhang et al. demonstrated that SH (4 mM) curtailed PTC cell proliferation, enhanced thyroid iodine-processing genes, and upregulated sodium/iodide symporter (NIS) expression via the thyroid-stimulating hormone receptor (TSHR)/cyclic adenosine monophosphate (cAMP) pathway, leading to improved radioactive iodine (RAI) uptake [50]. R.Z. Li et al. reported the bidirectional therapeutic effect of SIN on rheumatic arthritis (RA) and lung cancer. They found that SH was highly sensitive to H1819 at 50 µM and H1975 at 200 µM, inducing apoptosis in cancer cells by increasing p-AMPK and decreasing p-mTOR. Additionally, SH (0–400 µM) showed no cytotoxic effect on normal lung cells (CCD19-Lu). SH (25, 50, 100 mg/kg) inhibited NSCLC tumors by phosphorylating AMPK with therapeutic effects comparable to cisplatin but with lower toxicity [51]. The detailed aspects of the antitumor activity of sinomenine hydrochloride are summarized in Table 2.

### 3.3. Combination Strategies for Synergetic Enhancement between SIN and Other Drugs

Combination therapy is a prevalent approach in clinical settings to achieve enhanced therapeutic effects. In 2011, X.M. Tong et al. reported that the combination of SIN (15.2–60.7 nM) and aclarubicin (ACLA) (117.9 nM) significantly augmented ACLA-induced apoptosis and reduced prostaglandin E2 (PGE2) production [52]. In 2010s, Liao et al. announced that SIN (20–80 µM) combined with 5-fluorouracil (5-FU) synergistically inhibited the proliferation of gastric cancer cells. The concurrent use of SIN and 5-FU proved more effective than their singular administration, without notable side effects, and the caspase-3/-9 cascade played a role in this synergistic effect [53]. Eight years later, J.J. Cao and team declared that SIN (3.9 mM) combined with 5-FU (44.92 mM) synergistically enhanced the inhibition of proliferation in hepatocellular carcinoma HepG2 cells [54]. Drug resistance, particularly P-gp-mediated resistance, is a frequent cause of chemotherapy failure [55]. In 2014, Z. Liu et al. reported that SIN (500 mM) boosted the cytotoxic effect of adriamycin on Caco-2 and multidrug resistance (MDR)-Caco-2 cells. SIN decreased PGE2 in MDR-Caco-2 cells and inhibited P-gp/MDR1 and COX-2 by obstructing the NF-κB pathway [56]. The following year, X. Liu. and co-workers reported that SIN (50–400 µM) counteracted cisplatin (3.8–16.8 µM) resistance in A549 cells. The combination of SIN and cisplatin increased miR-200a-3p by directly targeting glutaminase, leading to the suppression of glutamine metabolism and increased sensitivity of lung cancer cells to cisplatin [57]. Combination strategies are also crucial in other contexts. For instance, early in 1999, B.H. Vieregge et al. conducted biological evaluations of SIN combined with immunosuppressive drugs. Their results indicated that SIN inhibited human lymphocyte proliferation when combined with tacrolimus and mycophenolic acid [58]. In 2014, Y. Sun et al. explored SIN’s combination with methotrexate (MTX) in treating arthritis in rats. They discovered that the combination of SIN (120 mg/kg) and MTX synergistically reduced synovial inflammation and joint damage in collagen-induced arthritis (CIA) rats by upregulating osteoprotegerin (OPG), downregulating RANKL, and increasing the OPG/RANKL ratio. The combination of SIN (303.6 μM) and MTX diminished RANKL and RA-FLS while elevating OPG in vivo [59]. The comprehensive aspects of synergistic enhancement strategies combining SIN with other drugs are summarized in Table 3.

### 3.4. Antitumor Activity of SIN Derivatives

**Figure 7:**
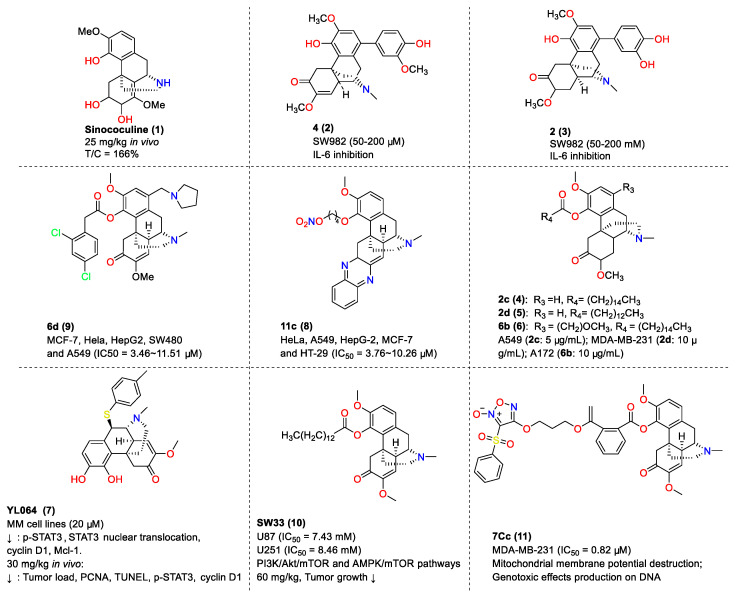
Antitumor activity of SIN derivatives. ↓: Decrease or inhibition.

In addition to SIN, numerous SIN derivatives have been investigated for their potential as bioactive agents. Early in 1995, Y.K. Hitotsuyanagi et al. first reported the effectiveness of the SIN derivative sinococuline (**1**) as an anti-cancer compound at 25 mg/kg (T/C = 166%), with its chemical structure displayed in Figure 7 [60]. Subsequently, two years later, Z. S. Deng’s group found that SIN derivative **4** (**2**) (50, 100, 200 µM) displayed a superior inhibitory effect on IL-6 production in SW982 cells compared to SIN and other derivatives [61]. Z.S. Deng et al. also reported that SIN derivative **2** (**3**) exhibited 83%, 95%, and 96% inhibition of IL-6 at a concentrations of 50 mM, 100 mM, and 200 mM, respectively, higher than SIN’s 12% inhibition at 200 mM [62]. Six years later, C.J. Wei et al. completed the synthesis and antitumor evaluation of SIN derivatives modified at the C-1, 4, 7, and 8 positions of SIN. They identified derivatives **2d** (**5**) and **6b** (**6**) with inhibition rates of 79.72% and 84.54% against MDA-MB-231 and A172 cells, respectively, at 10 µg/mL. The inhibition rate of compound **2c** was 80.49% against A549 at 8.8 µM, displaying superior antitumor activity compared to the positive control 5-FU [63]. Around the same time, Y.Y. Wang et al. demonstrated that the SIN derivative **YL064** (**7**) selectively induced death in multiple myeloma (MM) cells and primary MM cells by inhibiting STAT3 (Tyr705) at 20 µM. It could bind directly to the SH2 domain of STAT3, inhibiting STAT3 dimerization. At a dosage of 30 mg/kg, it significantly reduced tumor load and affected PCNA, TUNEL, p-STAT3, and cyclin D1 in vivo [64,65].

In 2021, researchers reported that the highly active compound **6d** (**8**) exhibited notable cytotoxicity through the PI3K/AKT and MAPK signaling pathways, with IC_50_ values ranging from 3.46 µM to 11.51 µM against various cancer cell lines (MCF-7, HeLa, HepG2, SW480, and A549). However, compound **6d** demonstrated less selectivity towards the normal cell line Hek293, with an IC_50_ of 3.46 µM [66]. G.H. Du et al. found that the SIN ester derivative **SW33** (**9**) exhibited anti-glioblastoma multiforme (GBM) potency with IC_50_ values of 7.43 mM and 8.46 mM against U87 and U25, respectively. It led to G2/M phase blockade and induced mitochondria-dependent apoptosis and autophagy through the PI3K/Akt/mTOR and AMPK signaling pathways. When administered at 60 mg/kg, it also displayed antitumor activity in vivo without significant systemic toxicity in major organs and blood [67]. A year later, Q.Z. Hu and others discovered that SIN derivatives **11c** (**10**) displayed pronounced cytotoxicity through the IL-6/PI3K/Akt and NF-κB signaling pathways, with IC_50_ values of 3.76–10.26 µM against cancer cells (HeLa, A549, HepG-2, MCF-7, and HT-29) [68]. In the same year, X. Gao et al. found that the SIN derivative **7Cc** (**11**) exhibited anti-breast cancer potency with IC_50_ values of 1.75 and 0.82 µM against MCF-7 and MDA-MB-231, respectively, and the second-highest cytotoxicity against A549 (IC_50_ = 1.94 µM). Moreover, **7Cc** (**11**) had the highest selective index (SI) values for MCF-7 and MDA-MB-231 cells (15.73 and 33.57, respectively) compared to the normal human cell line MCF10A [69].

## 4. Anti-Inflammatory Activity and Analgesic Activity

### 4.1. Anti-Inflammatory Activity and Analgesic Activity of SIN

Inflammation is the body’s defensive response to external or internal stimuli and a critical aspect of the body’s resistance function. Many natural products have demonstrated anti-inflammatory potential [70]. Early in 1996, L. Liu et al. reported that SIN (150 mg/kg) reduced joint swelling and the erythrocyte sedimentation rate (ESR) and inhibited the proliferation of rat synovial fibroblasts [71]. X.B. He and team found that SIN (0.1–5 mM) inhibited macrophage proliferation and caused the nuclear fragmentation, aggregation, and condensation of Mouse mononuclear macrophage leukemia cells Raw264.7, inducing macrophage apoptosis through increased p27 and Bax and decreased BcL-2, partially via ERK activation [72]. T.W. Kok et al. showed that SIN (125–1000 µM) arrested the HUVEC cell cycle in the G1 phase, inhibited chemotaxis, and reduced HL60 cell migration across HUVEC monolayers at 100 µM [73]. In the same year, Y. Wang and team reported that SIN (91.1–36.4 µM) inhibited TNF-α and IL-1β in peritoneal macrophages (PMs) and synoviocytes, restrained NF-κB, and increased IκB, demonstrating anti-inflammatory efficacy [74]. In 2005 and 2006, H.L. Zeng et al. reported that an N-oxide of SIN had the highest inhibitory effect on NO release (IC_50_ = 23.04 µM) compared to a positive control L-NMMA (IC_50_ = 28.03 µM) [75,76]. H. Cheng and team found that SIN (100 mg/kg and 200 mg/kg) alleviated colitis in mice by reducing TNF-α and interferon-γ (IFN-γ) [77]. A.L. Wang et al. reported that SIN (0.1 and 1 mM) inhibited advanced glycation end products (AGEs)-induced release of TNF-α, IL-1β, and IL-6 from retinal microglia and reduced ROS production and the nuclear translocation of NF-κB-p65, leading to inhibition in retinal microglia cells in rats [78].

In 2008, M.H. Wang et al. reported that SIN (1, 10 µM) increased opioid µ-receptor (OMR) phosphorylation in Chinese hamster ovarian (CHO) cells and displayed analgesic effects by activating OMR in mice at doses of 10–30 mg/kg [79]. H. Zhou’s group found that SIN (100 mg/kg) inhibited the incidence and progression of CI. In addition, SIN improved arthritis in rats by inhibiting pro-inflammatory cytokines IL-1β and IL-6, inhibiting MMP-2/-9, and increasing TIMP-1/-3 [80]. Y. Cheng et al. declared that SIN (240 mg/d) reduced albuminuria and increased complement C3 levels by decreasing T-β mRNA, the T-β/GATA-3 ratio, and IFN-γ without significantly inhibiting the Th2 pathway, thereby regulating the Th1/Th2 balance [81]. B.D. Huang and colleagues reported that SIN (1–5 mM) inhibited MMP-1/-3/-9/-13 in SW1353 cells and human osteoarthritis chondrocytes treated with IL-1, blocking IL-1β-induced catabolism via proteolytic enzyme inhibition [82]. X.D. Ju et al. found that SIN (10–250 mM) inhibited IL-1β-induced glycosaminoglycan (GAG) release, reduced MMP-13, increased TIMP-1, decreased chondrocyte death, and blocked IL-1β-induced chondrocyte apoptosis by inhibiting DNA fragmentation and caspase-3 activation [83]. In 2011, Y.Q. Ou and co-workers reported that SIN (0.01–1.00 mM) inhibited the invasion and migration of fibroblastoid synovial cells (FLS) co-cultured with THP-1, decreasing CD147 and MMP-2/-9 [84]. D.P. Chen et al. found that SIN (75.9–607.2 µM) inhibited the TNF-α-induced expression of vascular cell adhesion molecule (VCAM-1), inflammatory factor IL-6, and chemokines CCL2 and CXCL8 in normal and fibroblast-like synoviocytes. SIN blocked TNF-α-induced NF-κB activation by downregulating p-IκBα and combined with LMS to further downregulate p-IκBα and p-ERK in FLS [85]. In 2021, Y.C. OH and team found SIN to be cytotoxic to HMC-1 cells (IC_50_ = 52.73 µM) and reduced TNF-α, IL-6, IL-8, and COX-2 at 10 and 20 µM [86].

In 2013, X.J. Li et al. demonstrated that SIN (80 mg/kg) effectively reduced hind paw swelling and bone loss, increased body weight, and lowered serum TRACP5b and receptor activator of NF-κB ligand (RANKL) levels. It was observed to diminish the phosphorylation of p38 (p-p38) and JNK (p-JNK), lessen calcium influx, impede the activation of NFATc1 and AP-1, and decrease the expression of *Fra-1* and *Fra-2* genes as well as c-Fos protein [87]. Additionally, Q. Yu and team found that SIN (100 and 200 mg/kg) enhanced the weight and survival rate of colitis mice, diminished MPO activity, and downregulated miR-155, c-Maf, TNF-α, and IFN-c [88]. H. Mu et al. discovered that SIN (100 mg/kg) alleviated symptoms in AIA rats by reducing the expression of MyD88, TLR2/TLR4, TNF-α, IL-1β, and IL-6 in their synovial tissue [89]. Q. Zhu and team observed that SIN (10–40 mg/kg) had an analgesic effect through targeting GABA_A_ receptors [90]. In 2015, L. Yi et al. reported that SIN (100 µM) inhibited TNF-α and IL-6, raised cytoplasmic IκBα, and decreased nuclear NF-κB-p65 by suppressing α7nAChR in LPS-treated Mouse mononuclear macrophage leukemia cells Raw264.7 [91]. H.C. Zhang et al. noted that SIN (0.125–1 mM) lowered ALP activity, MyD88, and TRAF-6 at a dose of 0.5 mM [92].

Q. Zhu and team also found that SIN (5–80 mg/kg) exerted an analgesic effect on postoperative rats, unaffected by gender, without inducing tolerance, by targeting GABA_A_ receptors [93]. In the same year, H.R. Bao’s group discovered that SIN (25–75 mg/kg) reduced eosinophils and goblet cells in sensitized animals in vivo by lowering TGF-β1 and CTGF, and increased total lung antioxidant capacity [94]. B. Tong et al. demonstrated that SIN (120 mg/kg) decreased the arthritic index, paw swelling, inflammation, and bone erosion scores, significantly reduced serum total IgG and IgG2a, as well as IL-1β, TNF-α, IL-6, and IL-17 levels, and moderately increased IL-10 levels [95]. The following year, S.Q. Rao and others found that SIN (40 mg/kg) enhanced the mechanical withdrawal threshold (MWT, about 50%) and thermal withdrawal latency (TWL, about 80%) in T2DM rats and inhibited ATP activation currents in HEK293 cells transfected with P2X3 receptors. Further studies indicated that SIN decreased P2X3 expression in dorsal root ganglia of type 2 diabetes mellitus (T2DM) rats and downregulated p-P38MAPK, leading to the alleviation of pain behavior in T2DM rats [96]. H.F. Xiong and team reported that SIN microspheres, through inhibiting the DSS-induced activation of TLR/NF-κB signaling pathway, alleviated inflammatory response by decreasing IFN-γ, IL-1β, TNF-α, IL-12p70, IL-6, and increasing SIGIRR and IL-10 [97]. Y. Hu et al. found that SIN inhibited key control genes in LPS pathogenesis, including five upregulated genes (*ARG1*, *TLR2*, *IL1A*, VCAM1, *DKK3*) and five downregulated genes (*HABP2*, *ID1*, *CHDH*, *GPX3*, *PTGFR*), particularly targeting *IL1A* and *FMO3* [98].

F. Qin and team noted that SIN (25–100 µM) decreased TNF-α, IL-1β, IL-6, increased miRNA-183-5p, decreased SP1, p-p65, p50, and increased IκB-α [99]. W.W. Liu et al. found that SIN (50 and 100 mg/kg) inhibited cytokines, decreased the percentage of certain synovial and mononuclear/macrophages in CIA mice, and reduced RA activity and DAS28 score [100]. M.F. Yue et al. observed that SIN selectively enhanced the production of vasoactive intestinal peptides (VIP) in gut and neuronal cells of CIA rats via the α7nAChR-PI3K/Akt/mTOR pathway, improving systemic inflammation [101]. Y. Yuan and team reported that SIN (30 mg/kg) increased MWT, TWL, and frequency response to cold stimulation, reduced TNF-α, IL-1β, and IL-6 in inflammatory pain (IP) rats, alleviated inflammatory pain induced by complete Freund’s adjuvant (CFA), and inhibited p-p65 and p-p38 through the suppression of p38 MAPK and NF-κB activation as well as COX-2 and PGE2 in IP rats [102]. T.W. Kim et al. found that SIN (50 and 100 mg/kg) decreased inflammatory cells, inhibited protein leakage, downregulated TNF-α and PGE2, and impeded NF-κB-p65 subunit translocation to the nucleus through the inhibition of NF-κB associated pro-inflammatory cascade [103]. M.M. Xu and co-workers reported that SIN (50 mg/kg) plus acupuncture reduced TNF-α, IL-6, IL-1β, and IL-8, increased superoxide dismutase (SOD) and malondialdehyde (MDA), and inhibited COX-2, iNOS, MMP-2, and MMP-9 in arthritis rats as well as nuclear factor κB and phosphorylated p38 mitogen-activated protein kinase (MAPK) [104].

**Figure 8:**
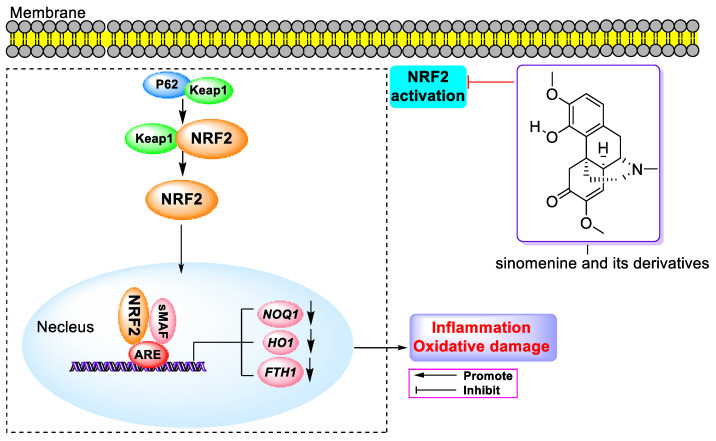
Keap1/NRF2 pathway related to the anti-inflammation and anti-oxidation activities of sinomenine and its derivatives.

Y.Z. et al. demonstrated that SIN (100 mg/kg) notably reduced body weight and DAI score, ameliorated colon shortening in mice with DSS-induced colitis via activating the Nrf2/NQO-1 pathway [105]. J. Shen and team found that SIN (0.25–1 mM) mitigated BK-induced inflammation in MG-63 cells by downregulating IL-1β, IL-6, TNF-α, p-p38, and p-NF-κB-p65 in the P38 MAPK and NF-κB signaling pathways, reduced MDA, increased SOD and CAT, and upregulated Nrf2, HO-1, and NQO-1 via the inhibition of Nrf2 pathway activation (Figure 8) [106]. S.P. Chen and team reported that single and repeated administrations of SIN (10–40 mg/kg) alleviated mechanical hypersensitivity in rats with cancer-related bone pain, inhibited microglial activation via the JAK2/STAT3 pathway, and decreased CAMKII/CREB through the inhibition of microglial JAK2/STAT3 and neuronal CAMKII/CREB cascades [107]. J.Q. Jiang et al. concluded that QingFengTeng and GuiZhi extracts significantly lowered IL-1β, IL-6, and TNF-α in the serum of AA rats and increased IL-10, demonstrating the extract’s (4:1, 1.5 g/kg) superiority over the compared groups [108].

Y.F. Wu and co-workers reported that SIN (10 mg/kg) increased the thickness of articular cartilage and inhibited the degradation of ECM in OA mice by suppressing NF-κB activity through the activation of the Nrf2/HO-1 signaling pathway [109]. Y. Wang and team found that SIN (30 µM) enhanced cell viability, inhibited apoptosis, reduced the production of ROS, and decreased IL-6 and TNF-α through the NF-κB and MAPK signaling pathways [110]. H. Song et al. demonstrated that the SH antioxidant surface transporter (AS-TE) alleviated joint swelling, decreased bone defects, and lowered TNF-α, IL-6, ROS levels, and ESR in RA rats [111]. Y.X. Liu and team observed that SIN (1.0 µM) blocked lipopolysaccharide (LPS)-induced apoptosis, diminished cleaved caspase-3/-9, and reduced IL-6, TNF-α, COX-2, and iNOS levels in HaCaT cells. They also found that SIN inhibited the phosphorylation of p65, IκBα, and p38MAPK by blocking the LPS-induced activation of NF-κB and MAPK and suppressing the production of colon-cancer-associated transcript-1 (CCAT1), resulting in anti-inflammatory effects [112]. C.P. et al. reported that SIN (120 mg/kg) reduced paw volume, AI, TNF-α, and ESR by inhibiting α7nAChR in AIA rat tissue cells, leading to an anti-arthritis effect [113]. R.L. Zhu and co-workers delineated that SIN (300 µM) inhibited the production of TNF-α, MCP-1, MIF, and MMP-9, decreased CD14 and TLR4, inhibited the release of intracellular Ca^2+^, downregulated NF-κB activation, and increased STAT3 phosphorylation in LPS-stimulated Mouse mononuclear macrophage leukemia cells Raw264.7 by decreasing CD14, TLR4, and intracellular free Ca^2+^ in macrophages, activating the JAK2/STAT3 pathway, and inhibiting inflammatory response [114].

G.A. Barr et al. reported the analgesic effects of SIN on infant rats in 2020. Their studies showed that SIN (0, 20, 40, and 80 mg/kg) produced moderate analgesic effects in both acute heat tests and inflammatory formalin tests [115]. M.Y. Zeng’s group found that SIN (100–1000 µg/mL) inhibited chemotaxis and secretion functions of LPS-stimulated macrophages, downregulated inflammatory cytokines (e.g., TNF-α, IL-1β, and IL-6), TLR4, MyD88, and p-IκB in the TLR4 pathway, and reduced NF-κB-p65 in the nucleus by blocking the TLR4/NF-κB signaling pathway [116]. Z.W. and others reported that SIN (3–75.9 µM) significantly reduced TNF-α, IL-1β, and IL-6 and enhanced SOCS in Mouse mononuclear macrophage leukemia cells Raw264.7 treated with LPS. SIN reduced the macrophage inflammatory response by downregulating miR-155 and upregulating SOCS1, leading to the inhibition of NF-κB transcription [117]. Y.Z. Du et al. reported that SH-loaded thermosensitive liposomes (SIN-TSL) combined with microwave thermotherapy showed promise in RA treatment. Their results indicated that the SIN-TSL (particle size 116.3 ± 5.03 nm) sustained-release system had good storage stability and compatibility. SIN-TSL targeted RA sites and prevented the systemic leakage of SIN. The complete release of SIN was achieved under microwave hyperthermia stimulation at RA sites, providing an anti-RA effect [118]. W. Qi et al. reported that SIN exhibited anti-arthritis biopotency, inhibiting MMP production by increasing SOCS3 and impeding IL-1β-induced TRAF6–TAK1 interaction and IL-6-induced JAK2 and STAT3 phosphorylation [119]. Also, in 2020, H. Wei et al. reported on the metabolic mechanism and anti-inflammatory effect of SIN and its main metabolites. Their findings indicated that SIN and its major metabolite (SINO) played significant anti-inflammatory roles. SINO (10 µM) induced ROS production, and SIN (10–200 µM) increased IL-6, TNF-α, and NF-κB nuclear translocation in LPS-treated Mouse mononuclear macrophage leukemia cells Raw264.7 [120]. Y.F. Leng and team found that SIN alleviated inflammation in dorsal root ganglia, demonstrating that SIN (800 µM) inhibited the TNF-α-induced apoptosis of DRG cells, increased cell viability, and decreased ROS and lactate dehydrogenase (LDH) release. Mechanistic studies showed that SIN reduced P38MAPK, CREB, c-fos, p-CAMKII, NF-κB, COX2, TLR4, IL-1β, and IL-17A in TNF-α-induced DRG cells and spinal cord tissues of spinal nerve ligation (SNL) rats by inhibiting the p38MAPK/CREB signaling pathway [121]. W.C. Xu et al. reported that the anti-RA mechanism of SIN at concentrations of 0.3–30 µM appeared to be independent of its direct effects on T cells [122].

In 2021, N. Wu et al. reported that SH (35 ppm) increased the intestinal villus height and reduced intestinal inflammation in adult fish by inhibiting TNF-α and enhancing IL-10, IL-22, and FOXP3a. SH improved the dysregulation of the microbiome by inhibiting the aggregation of immune cells through glucose metabolism, thereby enhancing the function of the intestinal immune barrier [123]. N. He and team found that SIN (20 and 40 mg/kg) reduced TNF-α, IL-1β, and IL-6, decreased receptor-interacting serine/threonine kinase 3 (RIP3), p-JNK, and c-Fos, and increased the survival of neurons in the spinal dorsal horn [124]. L. Liu et al. reported that SIN (25–100 mg/kg) inhibited the migration of Mouse mononuclear macrophage leukemia cells Raw264.7 to the foot, reduced foot swelling, and decreased TNF-α and IL-6 expression in mice. SIN (160–640 µM) inhibited migration of LPS-treated Mouse mononuclear macrophage leukemia cells Raw264.7 and bone-marrow-derived macrophages (BMDMs), inhibiting activation of the SRC/FAK/P130CAS axis by reducing iNOS/NO production, and decreasing integrin αV and β3, thus directly inhibiting macrophage migration independent of its anti-inflammatory effects [125]. They also found that SIN (25–100 mg/kg) decreased the morbidity of CIA mice, reduced the swelling of the hind paws, decreased IL-6, IL-17, IL-1β, and TNF-α, and normalized the indexes of BV, BV/TV, BS, BS/BV, BS/TV, BMD, and TBMD. In addition, SIN (12.5–100 μM) inhibited IL-6, IL-33, and ROS production in TNF-α-treated RASF. Mechanistic studies suggested that SIN enhanced Nrf2 expression and nuclear localization by phosphorylating p62 at Ser351, leading to Keap1 degradation, and upregulated HO-1 by phosphorylating p62 at Thr269/Ser272, thereby exhibiting anti-arthritis effects [126].

In 2021, Y.D. et al. reported that SIN (200 µM) inhibited MCP-1, IL-6, and vascular endothelial growth factor, increased A_2A_R in synovial tissue of AIA rats and FLSs, and inhibited the NF-κB pathway via α7nAChR, thereby alleviating arthritis. SIN (120 mg/kg) also decreased the arthritis index in vivo [127]. Local and systemic inflammation are characteristics of rheumatoid arthritis (RA), a chronic autoimmune disease. Therefore, anti-inflammatory treatment is a strategy for RA [128]. Y. Huang et al. reported that a combined RA treatment of SIN formed a coamorphous system with three nonsteroidal anti-inflammatory drugs (NSAIDs) such as indomethacin, naproxen, and sulindac, improving solubility and slowing drug release [10]. Y. Zhou et al. reported that SH alleviated colitis by inhibiting the activation of the NOD-, LRR-, and pyrin-domain-containing protein 3 (NLRP3) inflammasome. SH (100 mg/kg) reduced the disease activity index and spleen index in colitis mice, shortened the colon length, minimized histological damage, and improved the dynamic balance and diversity of the bacterial community. Additionally, SH decreased TNF-α, IL-6, and inducible nitric oxide synthase and increased IL-10 and arginase 1 [129]. Y. Liu and team reported on the treatment of SIN in children with pneumonia. Their results showed that SIN (5–20 µM) inhibited glutathione S-transferase M1 (GSTM1), decreased TNF-α, IL-1β, and MCP-1, and increased IL-10, thereby alleviating inflammation and apoptosis in lipopolysaccharide (LPS)-stimulated WI-38 cells [130].

In the current year, Z.M. Jiang et al. demonstrated that SIN reduced zonula occluden-1 (ZO-1), occludin, claudin-1/-2, IL-17/-6/-1b, and RORct in TNF-α-induced MH7A cells and increased IL-10 and FOX3 in Treg cells. In addition, SIN regulated NF-κB and MAPK phosphorylation via an aryl hydrocarbon receptor (AHR)-dependent mechanism, influencing the Th17/Treg balance. The levels of p-p38 and p-p65 within MH7A cells were reduced, while AHR and CYP1A1 were notably overexpressed [131]. L. Zhao et al. found that SIN (60 mg/kg) ameliorated LPS-induced alveolar injury by lowering TNF-α and IL-6. SIN (1 mM) elevated adenosine A_2_A receptor expression, activated the peroxisome-proliferator-activated receptor β/δ (PPARβ/δ) in macrophages, and facilitated nuclear translocation and transcriptional activity of PPARβ/δ. A combination of SIN (30 mg/kg) and adenosine A_2_A receptor agonist (CGS21680 (0.05 mg/kg)) proved more efficacious in treating ALI than when used singly [132]. R.Z. Li and associates observed that SH (0–400 µM) reversed the LPS-induced decline in RAW264.7 cell viability and reduced RA-FLS cell activity. SH also lowered TNF-α levels and curtailed the release of inflammatory factors from LPS-stimulated Mouse mononuclear macrophage leukemia cells Raw264.7. It enhanced the phosphorylation of the AMPK pathway and suppressed the activation of the NF-κB pathway. This resulted in decreased levels of TNF-α, IL-1β, and IL-6 in rats with adjuvant arthritis [51]. H. Jiang and team reported on the efficacy of SIN (90 mg/kg) in improving adjuvant arthritis. SIN (50 and 100 µM) managed LPS-induced inflammation through the MAPK and NF-κB signaling pathways, inhibited LPS-induced phosphorylation of neutrophil p65, and significantly reduced p-ERK and p-P38 levels [133]. It is well-recognized that inflammation is often intimately linked to the development and progression of cancer. Hence, the anti-inflammatory properties of SIN are also beneficial in mitigating the onset and progression of tumors [134]. A comprehensive summary of the anti-inflammatory activity of SIN is presented in Table 4.

### 4.2. Anti-Inflammatory Activity and Analgesic Activity of Compounds Derived from SIN

**Figure 9:**
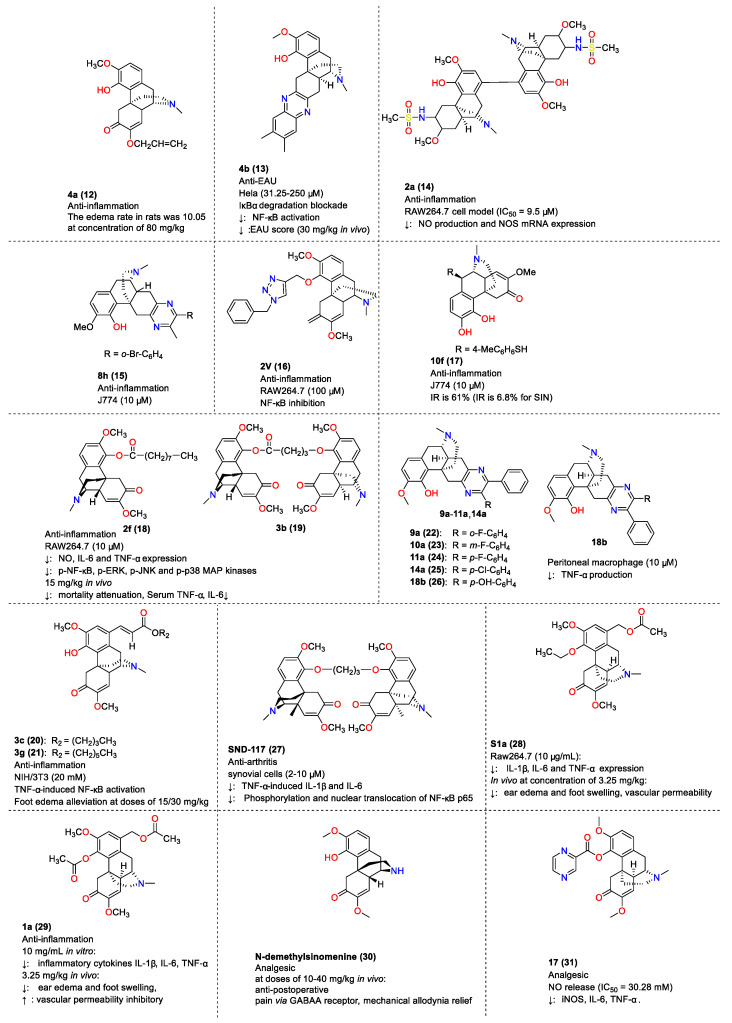
Anti-inflammatory activity and analgesic activity of compounds derived from SIN. ↓: Decrease or inhibition. ↑: Increase or induction.

Studies on the structural modification of SIN to obtain more active anti-inflammatory derivatives, such as those depicted in Figure 9, have been reported. For instance, Q. Tang et al. (2006) synthesized SIN C ring derivatives, identifying compound **4a** (**12**) as possessing the most potent anti-inflammatory activity. This compound reduced rat edema to a level comparable with aspirin at a dosage of 80 mg/kg [135]. Y.T. Lou et al. synthesized SIN derivatives and demonstrated their protective effect on experimental autoimmune uveoretinitis (EAU) in mice. Compound **4b** (**13**) exhibited half-maximal inhibitory concentrations (IC_50_) of 8.91 µM and 14.0 µM for T and B lymphocytes, respectively, and inhibited TNF-α-induced IκBα degradation and NF-κB activation in HeLa cells. At 30 mg/kg, it significantly reduced the EAU score and improved the condition of EAU mice [136]. In subsequent research, P. Teng et al. reported on the synthesis and evaluation of SIN derivatives. Compound **2a** (**14**) was found to be highly effective in inhibiting LPS-induced NO production in mouse macrophage Mouse mononuclear macrophage leukemia cells Raw264.7 with an IC_50_ value of 9.5 µM and an inhibition rate of 41.6% at 6.25 µM [137]. W. Meng et al. discovered that SIN derivative **8h** (**15**) significantly reduced TNFα production in LPS-stimulated mouse macrophages (J774) with an inhibition rate of 73% [138]. X.Y. Chai and others observed that most SIN derivatives were more effective than SIN itself in inhibiting LPS-mediated NF-κB activation, with compound **2v** (**16**) showing the most potent effect [139]. The introduction of a 10b-benzenesulfanyl group to SIN derivatives resulted in strong TNFα inhibitory activity, as demonstrated by compound **10f** (**17**), which exhibited more pronounced TNF-α inhibitory activity than SIN in LPS-stimulated mouse macrophages (J774) [140]. In 2012, P. Teng et al. also reported the synthesis and anti-inflammatory activity of SIN derivatives **2f** (**18**) and **3b** (**19**) (Figure 9), which decreased NO, IL-6, and TNF-α in Mouse mononuclear macrophage leukemia cells Raw264.7 without apparent cytotoxicity. Additionally, compound **3b** (**19**) specifically inhibited the phosphorylation of NF-κB and degradation of IκBα in the NF-κB signaling pathway, while **2f** (**18**) inhibited the phosphorylation of NF-κB, ERK1/2, JNK, and p38 MAP kinases. Both compounds reduced LPS-induced mortality in mice with septic shock, decreased serum TNF-α and IL-6 levels, and alleviated systemic inflammatory toxicity in vivo [141]. J. Jin’s group synthesized SIN derivatives, among which compounds **3c** (**20**) and **3g** (**21**) (20 μM) significantly inhibited TNF-α-induced NF-κB activation, with IC_50_ values for the cytotoxicity on mouse embryonic fibroblasts (NIH/3 T3) being 79.96 µM and 38.9 µM, respectively. Moreover, SIN derivative **3g** (**21**) significantly reduced carrageenan-induced foot edema in mice at dosages of 15 and 30 mg/kg [142]. T.T. Zhou et al. synthesized asymmetric pyrazin-SIN derivatives, finding that compounds such as **9a** (**22**), **10a** (**23**), **11a** (**24**), **14a** (**25**), and **18b** (**26**) were more effective than SIN in inhibiting TNF-α production in LPS-stimulated mouse peritoneal macrophages, with an inhibition rate exceeding 95% at a 10 µM dosage [143].

Y.R. Zhou et al. (2015) reported that SIN divalent **SND-117** (**27**) significantly mitigated erythema and swelling in mice, reduced bone erosion and joint destruction, and decreased IL-1β, IL-6, and TNF-α in the knee, besides inhibiting NF-κB-p65. SND-117 also inhibited TNF-α-induced IL-1β and IL-6 expression in a non-cytotoxic concentration range of 2–10 µM [144]. In the same year, Z.J. Zhao et al. reported the anti-inflammatory effects of a novel SIN derivative **S1a** (**28**), which significantly reduced IL-1β, IL-6, and TNF-α in Raw264.7 cells and inhibited ear edema and foot swelling in animal models without significant toxicity at concentrations below 20 µg/mL [145]. Subsequently, Z.J. Zhao et al. (2016) demonstrated that SIN derivative **1a** (**29**) showed notable transdermal penetration and anti-inflammatory effects, reducing ear edema and foot swelling in rats more effectively than SIN and other derivatives [146]. Y.T. Ou et al. (2018) reported the anti-pain effect of **N-demethyl SIN** (**30**) in post-operative mice, finding that it alleviated mechanical allodynia by targeting the GABA_A_ receptor without significant tolerance [147]. In 2021, Z.Y. Zhou et al. found that **N-demethyl SIN** (**30**) attenuated neuropathic and inflammatory pain in mice, which was more effective than that of SIN [148]. F. Gao et al. reported that SIN derivative **17** (**31**) significantly inhibited the secretion of the pro-inflammatory factor μnitric oxide in Mouse mononuclear macrophage leukemia cells Raw264.7, with an IC_50_ value of 30.28 µM and no cytotoxic effects at concentrations up to 100 µM [149]. E.E. Shults et al. synthesized SIN derivatives and found that compound **9** (**32**) significantly reduced pain response when administered at a dose of 2.5 mg/kg [150]. The detailed points of the anti-inflammatory and analgesic activities of SIN derivatives are summarized in Figure 9.

## 5. Neuroprotective Activity of SIN

L. Qian et al. reported that SIN, in micromolar concentrations (10^−6^ to 10^−5^M), protected DA neurons in rat mesencephalic glial cells and reversed the LPS-induced reduction in Th-IR neurons and loss of neuronal processes. SIN effectively reduced LPS-mediated superoxide and intracellular ROS by hindering the LPS-stimulated transport of p47^phox^ to the cell membrane and downregulating NO, iNOS, TNF-α, PGE2, and COX-2 in enriched microglia [151]. Additionally, at a concentration of 0.4 mM, SIN diminished ROS and NO and lessened inflammatory molecules such as TNF-α, IL-6, and MCP-1 in BV2 cells induced by amyloid β (Aβ). SIN enhanced cell vitality and decreased the number of TUNEL stained cells, thus protecting HT22 cells and primary hippocampal cells from the indirect toxicity of ADDL [152]. Y.Q. Yang and associates observed that SIN, administered at doses of 10, 30, and 50 mg/kg, expedited the recovery of motor ability and lessened brain edema post-traumatic brain injury (TBI). It reduced TUNEL-positive neurons, increased BcL-2, and reduced caspase-3. Additionally, it alleviated TBI-induced oxidative stress by reducing MDA, increasing GP_X_ and SOD activities, and promoting Nrf2 translocation from the cytoplasm to the nucleus, thereby activating the Nrf2-antioxidant response element (ARE) signaling pathways [153]. J. Qiu et al. discovered that SIN (10 and 20 mg/kg) inhibited OGD-induced NLRP3 inflammasome activation both in vivo (10 and 20 mg/kg) and in vitro (0.1–1.0 mM). SIN reduced the activation of astrocytes and microglia after MCAO, reduced NLRP3, ASC, caspase-1, and IL-1β, and inhibited IL-1β/-6/-18 and TNF-α via the AMPK signaling pathway [154]. H. Shi and team demonstrated that 1 mM of SIN attenuated M1 markers, encouraged M2 markers, and reduced microglia-mediated neuronal toxicity as well as neuron and microglia apoptosis. At 100 mg/kg, SIN inhibited microglia infiltration and activation in intracerebral hemorrhage (ICH) situations, enhanced M2 polarization, suppressed M1 markers, and diminished MMP-3/9 in ICH. Furthermore, SIN at 20 mg/kg reduced the brain water content and nerve damage in mice with ICH [155]. In the same year, J.H. Yoo et al. found that SIN (20 and 40 mg/kg) suppressed spontaneous activity in mice, shortened sleep latency induced by pentobarbital, and extended total sleep time. SIN reduced non-rapid eye movement sleep time and increased total sleep by enhancing Cl^–^ flow in hypothalamic neurons, activating glutamic acid decarboxylase (GAD 65/67), and increasing hypothalamic GABA subunits (α4, β1, β2, γ3) [156].

The following year, J.Y. Ou et al. reported that SIN demonstrated a protective effect against morphine dependence both in vivo (60 mg/kg) and in vitro (100 µM) through the N-methyl-D-aspartic acid receptor (NMDAR) 1/CAMKII/CREB pathway. SIN suppressed astrocyte activation, decreased cAMP and Ca^2+^ levels in SH-SY5Y cells, and inhibited p-NMDAR1/NMDAR1, p-CAMKII/CAMKII, and p-CREB/CREB [157]. B. Gao and team found that SIN (20, 40, and 80 mg/kg) displayed anticonvulsant and neuroprotective effects on PTZ-kindled epilepsy rats by inhibiting NLRP1 inflammasome IL-1β/-18/-6 and TNF-α [158]. In 2019, T. Sakurada et al. reported that an oral administration of SIN (80 mg/kg) inhibited formalin-induced licking and biting responses and suppressed the formalin-induced activation of dorsal spinal PERK1/2. Naloxone hydrochloride and β-FNA significantly reversed the SIN-induced ERK1/2 activation inhibition in the spinal cord, highlighting SIN’s blocking of ERK1/2 activation via the µ-opioid receptor [159]. Simultaneously, L.L. Zhang and team reported that SIN (40 mg/kg) reduced spinal cord edema, decreased the Bax/BcL-2 ratio and caspase-3 in neurons, inhibited IL-1β/-6 and TNF-α, and increased MDA in rats with spinal cord injury. SIN (10 µM) upregulated nuclear Nrf2 in PC12 cells, promoting the nuclear translocation of Nrf2 and inhibiting IL-1β/-6 and TNF-α to protect cells from damage [160]. The following year, S.K. Sharma et al. found that SIN (100 µM) inhibited astrocyte activation and protected β-amyloid-treated neurons from damage, reducing the production of ROS, NO, and inflammation-related cytokines (e.g., IL-12p70/-10/-6/-1β/-8) [161].

Y.B. Lin and associates reported that SIN (80 mg/kg) significantly reduced the morphine-induced CPP effect, downregulated TH and NR2B, and upregulated µ-opioid receptor (zfmor) and δ-opioid receptor (zfdor1 and zfdor2) in zebrafish brain [162]. Z. Kiasalari et al. found that SIN (100 mg/kg) decreased IL-1β/-6/-18/-17A and TNF-α and increased IL-10 in MS mice. It relieved neuroinflammation, demyelination, and axon damage and loss, reduced inflammasome NLRP3, ASC, and caspase-1, and enhanced myelin basic protein (MBP) activity, decreasing glial fibrillary acidic protein (GFAP) and Iba1 immune activity [163]. In 2021, F.F. Bi et al. reported that SIN (20 mg/kg) reduced brain pathological lesions and water content. SIN (50–200 µM) alleviated middle cerebral artery occlusion (MCAO)-related inflammation and oxidative stress by inhibiting TNF-α and IL-1β production, reducing NO, SOD, and GPx enzymes. It suppressed M1 markers (NOS2 and IL-6), and boosted M2 markers (Arg^–1^ and IL-10), activating the Nrf2 pathway and upregulating Nrf2, enhancing Nrf2 nuclear translocation, upregulating HO-1 and NQO1, and inhibiting p-IκBα and NF-κB nuclear translocation [164]. M. Roghani et al. observed that SIN (100 mg/kg) increased the new object recognition (NOR) index, improved short-term Y-maze alternations, raised dark avoidance latency in passive avoidance patterns, and reduced detection error and latency in the Barnes maze task by downregulating neuroinflammation and oxidative stress [165]. X.F. Hou and team reported that SIN (75.9 µMM) inhibited iNOS, increased ARG-1, promoted polarization from M1 to M2, inhibited TNF-α and IL-6, and increased IL-10 in RAW264.8 cells treated with IFN-γ and LPS. Additionally, SIN increased type II collagen and aggrecan, inhibited apoptosis, decreased ROS and MDA, increased SOD, and decreased Bax, caspase-3, MMP-2/-9, and iNOS while increasing BcL-2 in LPS-induced human nucleus pulposus cells (NPCs) [166]. X. Bao et al. reported that SIN (20 mg/kg) improved motor function in PD mice by inhibiting the PI3K/Akt/mTOR pathway and increasing the autophagy of dopaminergic neurons. It enhanced the survival of these neurons, increased Beclin1, the LC3-II/LC3-I ratio, and LC3B-positive neurons, and decreased P62 [167]. Recently, J. Chen et al. reported that SIN (1 mL/kg) alleviated diabetic peripheral neuropathic pain (DPNP) by reducing PTGS2, inactivating the inositol-requiring enzyme 1 alpha-X-box-binding protein 1 signaling pathway, and inhibiting microglia cell activation and inflammatory factor release [168]. C.J. Fu et al. found that SIN (50 and 100 mg/kg) eased microglia-mediated inflammatory responses by promoting Nrf2 expression and nuclear translocation, enhancing Nrf2 downstream factors HO-1 and NQO-1. It reduced cerebral cortex water content post-subarachnoid hemorrhage (SAH), decreased the apoptotic fraction, Bax, and CC3, and upregulated BcL-2. SIN curbed the activation of SAH-derived microglia and reduced inflammatory factors IL-1β/-6 [169]. These neuroprotective activities of SIN are detailed in Table 5.

## 6. Immunosuppression Activity

In addition to its recognized anticancer, anti-inflammatory, and neuroprotective effects, SIN and its derivatives have exhibited a range of other biological activities. In 1985, H. Hojo et al. reported that SH, administered at 30 and 100 mg/kg, reduced the anti-sheep red blood cells (SRBC) plaque-forming cells (PFC) response in spleen cells and suppressed the LPS-induced increase in spleen cell numbers. This study highlighted the immunomodulatory potential of SH [170]. Y.W. Chen et al. discovered that SIN, at a concentration of 303.6 µM, diminished the expression of B7-H1 and B7-DC on tubular epithelial cells (TECs) and enhanced IL-2 and IFN-γ production in co-cultured CD4^+^ T cells. These findings provide insights into the significant immunoregulatory properties of SIN [171]. L. Shu et al. explored the immunosuppressive effect of SIN on CD4^+^ T cells, demonstrating that SIN, at 0.1 and 1 mM concentrations, induced apoptosis in these cells. The mechanism involved blocking cell cycle progression from the G1 phase to the S phase and enhancing caspase-3 cleavage [172]. Additionally, in 2007, Y.Y. Zeng et al. reported that SIN, administered at doses of 50, 100, and 200 mg/kg, effectively reduced clinical scores and the percentage of initial weight loss in experimental autoimmune encephalomyelitis (EAE). SIN also reduced cellular infiltration, TNF-α, and IFN-γ production in the spinal cord, along with decreasing the CC chemokines RANTES, MIP-1α, and MCP-1. These findings suggest that SIN has therapeutic potential in the treatment of autoimmune disorders like EAE [173].

Y.W. Chen et al. reported that SIN (200 μg/mL) could promote the differentiation of monocytes into dendritic cells (DCs). It enhanced CD11a, CD32, and MR and decreased CD14, CD86, CD40, B7-H1, and HLADR in LPS-induced DCS. It also inhibited IL-12/-2, and IFN-γ production [174]. J.L. Huang et al. reported that SIN (0.5 and 1.0 M) inhibited VCAM-1 in TNF-α-induced HUVECs (no cytotoxicity was observed at a concentration of 0.25–2.0 M). This implied that SIN can serve as an immunotherapy modulator for rheumatic carditis or rheumatic heart disease [175]. Y. Zhao and co-workers reported that SIN (2 mM and 5 mM) decreased the HLA-DR, CD40, CD80, CD86, CD83, and CPM value. It also decreased IL-1, NF-κB, and p-IκBα and inhibited the migration of RelB from the cytoplasm to the nucleus. This implied that SIN has value in DCs-mediated autoimmune diseases [176]. In 2008, F. Huang and co-workers reported that SIN (0.5–2 mM) reduced high-affinity IgE receptors (FcεRI), stimulated RBL-2H3 to release β-amino-hexosamine, and inhibited IL-4 and TNF-α secretion and the phosphorylation of GAB2, Akt, and p38MAPK [177]. A. Kato et al. reported that SIN inhibited the L-histidine decarboxylase of Lactobacillus 30A in a non-competitive manner (IC_50_ = 969 mM, KI = 762 mM). This potency may influence the histamine synthesis and release within mast cells and basophils, thus modulating the immune and inflammatory response [178]. In 2015, authors found that SIN (2–31.25 mM) promoted the release of β-hexosaminidase within RBL-2H3 cells via phosphorylating CPLA2 and ERK, increasing ANXA1 cleavage and COX-2, leading to the enhancement of PGD2 and PGE2 release. These results were helpful in explaining the anaphylactic reaction of Zhengqing FongtongNing (ZQFTN), a pharmaceutical drug for rheumatoid arthritis and other autoimmune diseases, the major component of which was SIN [179]. In 2016, N. Wang et al. reported that SIN (0.1–100 μM) increased β-hexosaminidase release and decreased histamine release in P815 cells. In addition, SIN (100 μM) (cytotoxicity IC_50_ was 500 μM) promoted IP3 production and increased intracellular Ca^2+^ concentration, IP3R protein, PLCγ and p-Src, p-Lyn, and PLCγ. SIN (0.364, 1.82 and 9.10 mg/kg) increased mouse ear vascular permeability and increased IP3 and TNF-α release [180]. The detailed points of the immunosuppression activity of SIN are summed in Table 6.

## 7. Anti-Depression Activity of SIN

Depression, particularly in geriatric cases, commonly referred to as “mental flu,” constitutes a significant public health challenge. The onset of depression is often linked with various disorders such as hypertension, coronary heart disease, and diabetes, making its management crucial for enhancing quality of life [181]. Research has demonstrated the anti-depressant potential of SIN administered at dosages of 20 and 40 mg/kg. SIN has been shown to improve social interaction and sucrose preference in subjects. Moreover, at a dosage of 40 mg/kg, SIN notably increased levels of brain-derived neurotrophic factor (BDNF) in the hippocampus and cortex by 126.2% and 138.73%, respectively. It also elevated the levels of phosphorylated neurotrophic receptor tyrosine kinase (pTrkB) and phosphorylated cAMP response element-binding protein (pCREBP) [182]. In the same vein, S.B. Liu et al. reported that SIN, at dosages of 30, 100, and 300 mg/kg, mitigated symptoms of depression induced by chronic unpredictable mild stress (CUMS) in animal models. Additionally, SIN corrected the imbalance of hippocampal neurotransmitter levels caused by CUMS, significantly increasing the levels of NE and 5-hydroxytryptamine (5-HT) in the hippocampus of mice. It also decreased IL-1b/-6 and TNF-α and inhibited the activation of the p38MAPK-NF-κB pathway while concurrently increasing the levels of NLRP3, ASC, and caspase-1 [183]. These findings, detailing the anti-depression activity of SIN, are summarized in Table 7.

## 8. Anti-Sepsis Activity of SIN

In 2015, scientists discovered the anti-sepsis activity of SH. They found that an administration of 100 mg/kg SH attenuated cecal ligation and puncture (CLP)-induced organ damage in mice as well as urea nitrogen (BUN), creatinine (Cr), alamine transferase (ALT), and aspartate transaminase (AST). It increased the IL-6, TNF-α, LC3-II/LC3-I ratio in lung and liver, thus significantly increasing autophagosome formation in peritoneal macrophages (PM), and SH-induced autophagy activity was inhibited by 3-methyladenine, an autophagy inhibitor [184]. F.J. Huang et al. uncovered that SIN alleviated acute lung injury (ALI) in mice with sepsis. They reported that SIN (100 mg/kg) alleviated LPS-induced lung injury and decreased the wet/dry (W/D) ratio of lung tissue. In addition, SIN increased Nrf2 and HO-1 and decreased KEAP1 and NQO1, improved the oxidative stress and inflammation via decreasing IL-6 and TNF-α in tissues, increasing the activity of SOD and decreasing the content of MDA in serum, and increased autophagy-related proteins LC-3II, ATG5, and Beclin1 [185]. W.S. and co-workers reported that SIN can alleviate acute lung injury in mice with sepsis. SIN (50–400 μM) played a protective barrier function via increasing aromatic hydrocarbon receptor and CYP1A1 in Caco-2 cells, increasing Claudin1, activating Nrf2, and upregulating HO-1 and NQO-1 [186]. The points of the anti-sepsis activity of SIN are summed in Table 8.

## 9. Organs Protection

### 9.1. Kidney Protection

In clinical practice, organ damage is a common complication associated with many diseases, making the protection of vital organs a crucial aspect of medical care. In 2012, J. Zhang’s group conducted a study that demonstrated the protective effects of SIN on vital organs. They found that SIN, at doses of 10 and 30 mg/kg, significantly mitigated weight loss caused by doxorubicin (DOX), improved overall health conditions, and reduced the foot process width in rats. SIN also decreased urinary protein excretion, increased total protein and serum albumin levels, and reduced cholesterol and triglycerides. Additionally, SIN lowered DOX-induced TNF-α and IL-1b levels, enhanced the expression of nephrin and podocin, and increased PPAR-α [187]. Z.Q. Zhao et al. reported that SIN, at a dosage of 200 mg/kg, reduced serum CR, BUN, relieved renal histological damage, and decreased caspase-3 in IR mice. SIN inhibited inflammatory infiltration by reducing macrophage and neutrophil infiltration and lowering CD11 levels. It attenuated IR-induced inflammatory mediators by decreasing CXCL-10, ICAM-1, TNF-α, and IL-6. Additionally, SIN reduced p-IKK-β and the degradation of IκB-α by inhibiting NF-κB signal transduction and MAPK activation to reduce p-P44/42, p-JNK, and p-P3 [188]. X.H. Lyu and team found that SIN, at a dosage of 200 mg/kg, increased the SOD level, decreased the MDA level, and inhibited myeloperoxidase (MPO) activity in I/R rats. This led to the alleviation of inflammatory infiltration and renal cell apoptosis induced by I/R. In addition, SIN, at concentrations ranging from 0.1–50 µM, reduced apoptosis by upregulating miRNA-124 in H/R HK-2 cells [189]. These studies highlight the potential of SIN in protecting vital organs from damage in various pathological conditions.

Also in 2016, T. Qin et al. reported that SIN (25–100 μM) activated the Nrf2 signaling pathway and elevated HO-1 and NQO1 in renal HEK293 and Mouse mononuclear macrophage leukemia cells Raw264.7. SIN inhibited LPS-induced M1 markers (IL-6 and NOS2), while it enhanced TGF-β-induced M2 markers (ARG-1 and IL-10), thereby regulating macrophage polarization and inflammation. It also attenuated renal fibrosis and ameliorated E-Cadherin, α-SMA, fibronectin, IL-1β, and TNF-α [190]. T. Qin’s group found that 100 mg/kg of SIN alleviated renal tubular dystrophy via elevating E-cadherin and decreasing α-SMA and fibronectin. It increased HO-1, NQO1, and Nrf2 in the nucleus. In addition, SIN reversed the H_2_O_2_-induced inhibition of the antioxidant enzymes catalase (CAT) and SOD-2 via reducing TGFβ-induced ROS, and it ameliorated the reduced enzyme activities of total SOD and GPx in cultured cells and UUO kidneys, thereby balancing the oxidative stress associated with renal fibrosis. Taken together, SIN attenuated renal fibrosis via inhibiting the fibrotic cell signaling of TGFβ/Smad and Wnt/β-catenin via the activation of Nrf2 [191]. L. Zhang etc. reported that SIN showed promise in the treatment of diabetic nephropathy (DN). It (20 or 40 mg/kg) reduced the blood glucose of DN rats via downregulating IL-18/-1 in kidney tissue and increasing claudin-5. It also alleviated glomerular endothelial dysfunction in diabetic nephropathy via activating the C/EBP-α/Claudin-5 axis [192]. H.P. Gu et al. reported the therapeutic effect of SIN on renal fibrosis via increasing PIK3CB and pathway activation caused by TGF-β1. SIN increased miR-204-5P via regulating the BMSC-derived exosome, thereby blocking the progression of renal fibrosis. It decreased pro-inflammatory cytokines, inhibited M1-type polarization, and promoted M2-type polarization. In total, SIN regulated the PI3K/Akt pathway via influencing miR-204-5p in BMSC-exosome, thereby improving the process of renal fibrosis [193]. Authors also reported that SIN (5 mg/kg) improved kidney injury via decreasing the expression of HO-1, 4-HNE, and 3-NT and alleviated inflammatory responses via decreasing the expression of TNF-α, STAT3, and p-STAT3 and NF-κB p65 in the cytoplasm and nucleus. It also decreased renal cell apoptosis via decreasing p21, Bax, Noxa, PARP1, and caspase-3/-8, increasing BcL-2 and SIRT6, and reducing TUNEL-positive cells [194]. The detailed points of the kidney protection activity of SIN are summed in Table 9.

### 9.2. Osseous Tissue Protection

L.G. He et al. reported that SIN (0.25–2 mM) inhibited the survival of mature osteoclasts, but it did not inhibit the activity of undifferentiated Mouse mononuclear macrophage leukemia cells Raw264.7. It also reduced and destroyed actin rings. SIN induced the apoptosis of RAW264.7 cells derived from OCLs via activating caspase-3. This discovery showed that SIN was promising in the treatment of excessive bone resorption diseases [195]. L.G. He et al. reported that SIN (25, 50 and 100 mg/kg) reduced craniolysis. It (0.25–1 mM) inhibited the formation and survival of LPS-induced osteoclasts (no cytotoxicity) and inhibited Tracp, MMP-9, C-src, integrin αVβ3, CK, and TNF-α. It also reduced c-Fos, Fra-1, and Fra-2 via reducing Ca^2+^ influx and inhibited TLR4 and TRAF6 as well as downstream MAPK (p-p38), NF-κB, AP-1, NF-ATc1, and TLR4 [196]. SIN (0.25 mM) inhibited RANKL and osteoclast formation via regulating PGE2 in MSCs (SIN concentrations ranging from 0 to 250 mM did not significantly affect the activity of MSCs). In addition, SIN decreased prostaglandin E synthase 3(PTGES3) or PGE2 and RANKL and increased OPG [197].

### 9.3. Brain Tissue Protection

In 2014, Z. Yang et al. reported that SIN (0.1 mM and 1 mM) diminished TNF-α, IL-1/-6, and ROS levels and suppressed ICH-induced microglial activation by inhibiting NF-κB. SIN also reduced the migration of BV-2 microglia (with no evident cytotoxicity) and mitigated cerebral-hemorrhage-induced head injury by attenuating inflammation in microglial cells [198]. In 2016, J. Qiu et al. discovered that SIN impeded neuroinflammation by activating astrocyte DRD2 along the CRYAB/STAT3 pathway. This led to a decrease in IL-1β/-6/-18, TNF-α, and p-STAT3 and an increase in DRD2 and αB-crystallin (CRYAB) in astrocytes following MCAO [9]. Three years prior, R.S. Sharma et al. reported that a PAMAM-OH dendrimer combined with SIN (D-Sino) (151.8–910.8 μM) significantly reduced early acute inflammatory responses by decreasing TNF-α, IL-1β/-6, and CCL-3. Furthermore, oxidative stress markers (e.g., iNOS and NO) in LPS-mediated mouse macrophage (RAW264.7) activation were reduced by impeding NF-κB and its nuclear translocation. D-Sino (30 mg/kg) specifically targeted activated microglia/macrophages at brain injury sites [199]. The contributions of SIN in terms of its osseous tissue and brain protection activities are summarized in Table 10.

### 9.4. Cardiovascular Tissue Protection

L.H. Zhu et al. demonstrated that SIN (200 µM) impeded vascular smooth muscle cell (VSMC) dedifferentiation without cytotoxic effects by counteracting the suppressive influence of platelet-derived growth factor (PDGF)-BB on SMα-actin and smoothelin. SIN notably augmented the proportion of VSMCs in the G0/G1 phase by inhibiting the phosphorylation of ERK1/2 and p38, Akt, GSK3β, STAT3, and PDGFR-β in the MAPK signaling pathway triggered by PDGF-BB [200]. In 2016, Q. Yin et al. found that SIN (25–100 mM) hindered endothelial barrier dysfunction caused by HG and restored abnormal occlusive protein distribution. It also suppressed RhoA/ROCK activation, reduced cell permeability, enhanced occludin expression, decreased ROS levels, and activated Nrf2. Consequently, SIN impeded RhoA/ROCK signal transduction activation by stimulating Nrf2-mediated ROS reduction, thereby preventing HG-induced disruption of renal endothelial barrier function [201]. SIN (30, 60, and 120 mg/kg) was also identified by C. Jiang’s group as beneficial in preserving cardiac function in diabetes mellitus (DM) rats. It inhibited the activation of NF-κB and reduced IκB, TNF-α, IL-1, IL-6, and the infiltration of CD3^+^- and CD68^+^-positive cells. In essence, SIN enhanced the cardiac function of DM rats by inhibiting NF-κB activity and the production of inflammatory cytokines [202]. SIN alleviated cardiac hypertrophy by activating the Nrf2/ARE signaling pathway. It suppressed the expression of ANP, BNP, β-MHC, ROS, and MD, downregulated caspase-3 and Bax, and upregulated BcL-2 both in vivo and in vitro. Mechanistic studies indicated that SIN reduced apoptosis in rats [203]. In the same year, L. Li et al. reported the protective effect of SIN against isoproterenol-induced CH in mice by inhibiting NF-κB activation and reducing TNF-α and IL-1β, thus improving oxidative stress and inflammatory responses and ameliorating myocardial hypertrophy in mice [204]. C.H. Lu and associates described the protective effect of SIN on myocardial ischemia-reperfusion (I/R) injury in rats. Markers of myocardial injury and levels of C-reactive protein (Hs-CRP), MCP-1, TNF-α, IL-1β, and IL-6 were reduced, leading to the amelioration of I/R injury in rats [205]. The specific points of the cardiovascular tissue protection activity of SIN are summarized in Table 11.

### 9.5. Liver Protection

Early in 1994, Yoshikazu Kondo et al. elucidated the protective effect of SIN on hepatitis in mice. Their findings indicated that SIN (10–100 mg/kg) mitigated liver injury and curtailed the production of tumor necrosis factor (TNF) and ROS, thereby treating Galn/LPS-induced liver injury [206]. Three years earlier, H. Chen’s group demonstrated that SIN diminished oxidative stress and inflammation by suppressing the activation of the TGF-β/Smad pathway, leading to the relief of APAP-induced acute liver injury. SIN enhanced the inflammatory response by decreasing MDA and LDH activities, increasing SOD and GSH-Px activities, and reducing TNF-α, IL-1β, IL-6, NLRP3, ASC, caspase-1, IL-1β, and the activation of NLRP3 inflammasome [207]. Z.G. Yang et al. reported that SIN inhibited oxidative stress and inflammatory responses by promoting Nrf-2/HO-1 signaling activation, thereby ameliorating inflammation by increasing TNF-α, IL-6, and IL-8 and decreasing IL-10 [208]. Y. Li et al. demonstrated the protective effect of SH on liver injury induced by Pb. SH downregulated Caspase-3, Bax, IL-1β, TNF-α, NF-κB, NF-κB p65, and IκBα and upregulated BcL-2 in Pb-exposed mice. The mechanism suggested that SH enhanced anti-inflammatory properties by reducing oxidative stress and inhibiting the NF-κB signaling pathway, thus alleviating chronic Pb poisoning [209].

### 9.6. Respiratory Protection

SIN alleviated *E. coli*-induced oxidative stress through the Nrf2/Keap1/PKC pathway, decreased p-NF-κB-p65, and inhibited the activation of NF-κB, thereby weakening pro-inflammatory cytokines and inhibiting the *E. coli*-induced inflammatory response [210]. H.J. He et al. reported that SIN (607.2 mM) impeded 16HBe cell migration and suppressed the expression of MMP-7/-9 and vimentin. Additionally, SIN (35 and 75 mg/kg) reduced serum levels of specific IgE and IL-4 in ovalbumin-induced asthmatic mice, mitigated airway remodeling, lessened subepithelial collagen deposition, and inhibited EMT by downregulating TGF-B1 and Smad3 [211]. J.L. Ma and team observed that SIN (5000 mg/kg) decreased capsaicin-induced high cough sensitivity and reduced inflammatory cell infiltration in guinea pigs by downregulating SOX5 and by decreasing intracellular Ca^2+^ levels and the secretion of SP and neurokinin A (NKA), thus reducing the infiltration of inflammatory cells [212]. The comprehensive details of the liver and respiratory protection activities of SIN are summarized in Table 12.

## 10. Antioxidant Activity

Also in 2017, Hua Fan et al. found that SIN (5 µM) enhanced the survival rate of PC12 cells exposed to H_2_O_2_ and diminished the oxidative stress response. It activated the Nrf2 antioxidant system (as shown in Figure 8) and suppressed NOX activation by triggering ROS-dependent production, thereby improving the resistance of nerve cells to oxidative stress [213]. H.Y. Zhang and team reported that SIN (10 mg/kg) decreased lipid peroxidation (LPO) and elevated levels of GP_X_, total antioxidant capacity, glutathione S-transferase (GST), and SOD. It also reduced IL-1β, TNF-α, IL-6, and NF-κB and inhibited MYD88, NLRP3, TLR4, and NF-κB in gestational diabetes mellitus (GDM) rats [214]. H.M. Xia and associates described the antioxidant activity and sustained release properties of SIN-loaded liposomes-in-hydrogel. Their findings indicated that SIN had a significant clearance of DPPH (IC_50_ = 25.5 μM) and H_2_O_2_ (IC_50_ = 1.1 mM) and reduced MDA in organs. The DPPH scavenging rates of SIN and its three preparations were ranked as SIN > SIN-L > SIN-H > SIN-L-H. SIN-L, SIN-H, and SIN-L-H (0.6 mM) exhibited similar H_2_O_2_ clearance rates, with the drug release being slow and lower than that of SIN alone [215]. The comprehensive details of the antioxidant activity of SIN are summarized in Table 13.

## 11. Drug–Drug Interaction

In 2007, Y.M. Yao et al. investigated the effect of SIN on human cytochrome P_450_ activity. They demonstrated that SIN (50 µM) inhibited CYP2C19 and facilitated the elimination of mephentoin [216]. Z.L. Lu and team explored the effect of SH on drug absorption in the intestinal epithelium. They discovered that SH (0.5%, 1%, and 2% *w*/*v*) reduced the trans-epithelial electrical resistance (TEER) of the Caco-2 cell monolayer and increased the apical-to-basolateral transport of all tested compounds while decreasing the basolateral-to-apical transport of the P-gp substrate cimetidine. SH enhanced drug absorption in the intestinal epithelium by promoting tight junction transients for pericellular transport, inhibiting the active efflux of P-gp substrates and enhancing molecular stability [217]. Y.L. Li and associates reported that SIN increased the intestinal absorption of octreotide (OCT). SIN (0.5% *w*/*v*) boosted OCT absorption in the gut through the activation of the PKC signaling pathway, thereby improving the pharmacokinetic behavior of OCT in rats. SIN also reduced TEER and increased FD-4 flux in Caco-2 cell monolayers [218]. Additionally, in 2019, W.C. Xu et al. found that SIN enhanced the immunosuppressive effect of methylprednisolone (MP), decreasing the IC_50_ value of MP. It regulated the glucocorticoid receptor (GR) translocation in Jurkat T cells and normal PBMCs. The synergistic effect of SIN on MP immunosuppression was linked to the nuclear GR translocation [219]. The extensive details of the drug–drug interactions of SIN are summarized in Table 14.

## 12. Other Activities of SIN

H.K. Li et al. reported that SIN (20 and 40 mg/kg) impeded orthodontic tooth movement (OTM) and root resorption, enhanced alveolar bone structure, increased bone volume/total volume (BV/TV), and decreased trap-positive osteoclasts on the compression side. SIN at concentrations of 0.1 M and 0.5 M augmented ALP activity, facilitated the deposition of mineralized nodules, upregulated OPG, ALP, and RUNX, and diminished RANKL in periodontal ligament stem cells (PDLSCs) [220]. In the same year, it was published that SIN promoted skin flap survival. SIN (80 µM) enhanced eNOS expression, reduced oxidative stress, increased autophagy flux, lessened apoptosis, and encouraged angiogenesis. Mechanistic studies revealed that SIN decreased cell apoptosis, upregulated eNOS expression, and promoted flap survival by activating the PI3K/Akt pathway [221]. M. S. Fan et al. reported that SH (25, 50, and 100 µM) inhibited BcL-2 and increased Bax protein expression, thereby suppressing the proliferation of BPH-1 cells. SH at doses of 0.5, 1, and 2 mg/kg decreased steroid 5-alpha reductase 2 (SRD5A2), PCNA, BcL-2, and MMP-2 [222]. The various other bioactivities of SIN are detailed in Table 15.

## 13. Other Bioactivities of SIN Derivatives

**Figure 10:**
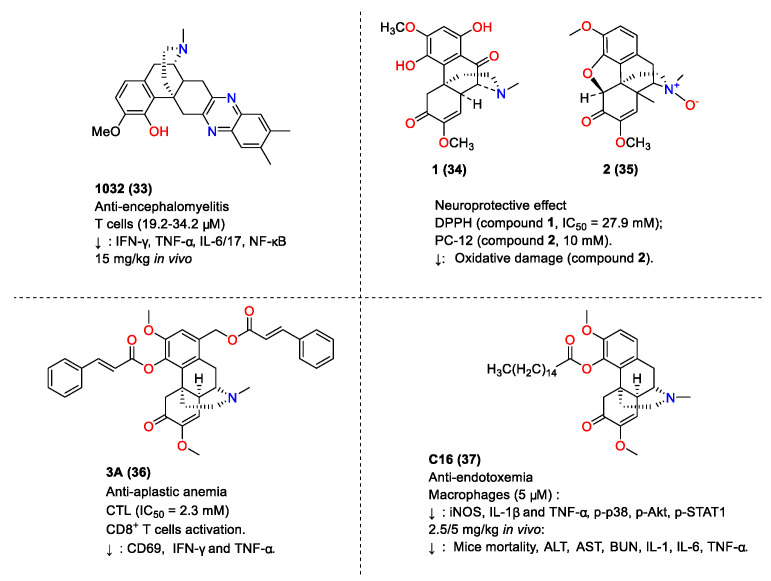
Other bioactivities of SIN derivatives. ↓: Decrease or inhibition.

L.C. Yan’s group reported the anti-encephalomyelitis effect of SIN derivative 1032 (**33**) (structure is shown in Figure 10). It (19.2–34.2 μM) inhibited the proliferation of T cells, decreased the production of IFN-γ, TNF-α, and IL-17, and inhibited TH17 differentiation via RoR-CT. It also inhibited the degradation of IκB-α, leading to the decrease in IL-6 production in bone-marrow-derived DC (BMDC) [223]. X.L. Wang and co-workers reported neuroprotective effect of SIN derivatives on microglia cells. They found the IC_50_ of compound **1** (**34**) in a DPPH inhibition assay was 27.9 mM. Another compound **2** (**35**) (10 mM) displayed neuroprotective effects by inhibiting the oxidative damage induced via β-amyloid_25-35_ in PC-12 cells [224]. The authors reported that SIN derivative **3A** (**36**) showed an obvious inhibitory effect on CFLL-2 (IC_50_ was 2.3 mM), and the inhibitory rate of NO production on macrophages was 97.1%. It can alleviate hematopoietic cell damage via reducing the activity of stimulated CD8^+^ T cells, reducing cell surface antigen CD69 expressed via activating T cells, impair aerobic glycolysis, and inhibit the release of IFN-γ and TNF-α in mouse CTL line CFLL-2 [225]. P. Ni and co-workers reported that SIN derivative **C16** (**37**) (2.5 and 5 mg/kg, no toxic effects on the heart, spleen, liver, kidney, and lung of endotoxin model mice) effectively reduced LPS-induced mortality, decreased ALT, AST, and blood BUN, and downregulated levels of IL-1β, IL-6, and TNF-α in mice. It (5 μM) also improved the LPS-induced apoptosis of macrophages, reduced iNOS, IL-1β, TNF-α, p38, Akt, and STAT1, and participated in the activation of ERK1/2, thereby reprogramming macrophages from M1 to M2 [226]. The detailed points of the other bioactivities of SIN derivatives are summed in Figure 10.

## 14. Conclusions or Concluding Remarks

Sinomenine and its derivatives have demonstrated a broad spectrum of biological activities, predominantly in the anti-inflammatory, antitumor, neuroprotective, and immunosuppressive domains. Although it is selected to treat acute arthritis and rheumatoid arthritis, clinical trials of other bioactivities (such as anti-prostate-cancer activity and neuroprotection) are needed. The primary pathways implicated in these activities include NF-κB, MAPK, Nrf2, PI3K/Akt/mTOR, and JAK/STAT. These findings underscore sinomenine’s potential as a valuable scaffold in drug design and modern drug discovery. Although recent advancements have been made in sinomenine research, there remain areas necessitating further investigation. Currently, sinomenine’s clinical applications are mainly limited to treating various rheumatoid diseases, with other disease treatments still in the basic research phase [227]. The direct pharmacological targets of sinomenine in terms of its anti-inflammatory, antitumor, and immunosuppressive effects are not fully understood, warranting more exploration of its potential molecular targets and antitumor and pharmacological mechanisms both in vivo and in vitro. Techniques such as chemical proteomics [228], photoaffinity chromatography [229], and drug affinity responsive target stability assays [230] can be utilized to investigate the intrinsic mechanisms of sinomenine and its derivatives, offering scientific explanations for any toxic side effects associated with certain derivatives [231].

The clinical application of sinomenine is constrained by its short half-life, high required concentration and low drug utilization [10,14,232]. Efforts to enhance sinomenine’s absorption rate and sustained release performance, such as incorporating it into hydrogels, are still in the nascent stages and require further validation [215]. Currently, sinomenine is administered primarily through injections, capsules, tablets, and patches [233], with limited research being conducted on improving drug absorption based on sinomenine derivatives. Therefore, developing new drug formulations or modifying sinomenine’s structure to enhance its drug utilization and clinical application is a promising research direction.

Sinomenine has demonstrated considerable efficacy in drug assistance reversal, though this area remains underexplored. Specifically, sinomenine has been shown to diminish the sensitivity of cisplatin to antitumor agents, reverse multidrug resistance, and significantly enhance antitumor efficacy [57]. When combined with indomethacin, naproxen, sulinic acid, and other non-steroidal anti-inflammatory drugs to form a common amorphous system, it enhances solubility, sustains drug release, and aids in the combined treatment of rheumatoid arthritis [10]. It is believed that the exploration of adjuvant drugs represents a promising avenue for future research. Furthermore, several newly reported sinomenine derivatives exhibit more significant biological activities than sinomenine itself, potentially targeting novel biological pathways [63]. This research holds promise for advancing treatments for diseases where significant progress is yet to be made. Given sinomenine’s broad spectrum of biological activities, its derivatives might possess diverse biological functions, many of which remain to be elucidated. For instance, the antitumor activity of sinomenine derivatives could suggest enhanced anti-inflammatory properties and vice versa [61]. Additionally, sinomenine has been shown to exert anti-inflammatory effects on HaCaT cells by downregulating the expression of TNF-α and IL-6 and reducing the production of cleaved caspase-3/-9 [112]. Similarly, matrine, another natural compound, inhibits COVID-19 virus replication and promotes the apoptosis of infected cells by altering TNF-α and IL-6 levels and increasing caspase-3 expression [234]. This suggests that sinomenine may be a promising antiviral drug for future investigation. Sinomenine has also been effective in improving arthritis in AIA rats by inhibiting the TLR2/NF-κB signaling pathway [89], a mechanism similarly observed with matrine in alleviating conditions like *Staphylococcus-aureus*-induced endometritis [234]. Therefore, sinomenine’s impact on this pathway suggests its potential as a significant drug for fungal infection treatment. Sinomenine hydrochloride has also been found to alleviate sepsis induced by cecal ligation puncture (CLP) in BALB/c mice by inhibiting the production of various inflammatory factors like TNF-α [184], while curcumin has demonstrated efficacy against sepsis through pathways such as PI3K/AKT, NF-κB, TNF-α, and TGF-B1 and possesses bactericidal effects against various bacteria [235]. These findings underscore sinomenine’s potential application in disease-resistant pathogen research and warrant further exploration and development.

In conclusion, sinomenine and its derivatives present considerable research potential, particularly as anti-inflammatory and antitumor agents. The anti-inflammatory effects imply that SIN is promising in dealing with rheumatic arthritis. This area of study merits sustained effort. With the ongoing advancements in combinatorial chemistry, rational drug design, and chemical proteomics, it is anticipated that more sinomenine derivatives with potent biological activity and novel mechanisms of action will be discovered globally.

## Figures and Tables

**Table 1:** Antitumor activity of SIN when used alone.

Activity	Cell Type or Model In Vitro (Effective Concentrations or IC_50_ Values)	Mechanism of Action	In Vivo		Year	Ref.
			Dose (mg/kg)	Therapeutic Effect		
Cytotoxicity	IL-1β-activated Hs701.T (IC_50_ = 0.125 mM)	↓: JAK3, EDG4, IL-13, PCTAIRE-3, ERF-1, HHR6A, HSP27, Daxx, TNF-A, COL1A2, IL-6, SATB, IFITM1, TNFRII, JAG2, MMP-13, and PLG.	NR		2006	[19]
Invasion and migration inhibition	THP-1 (effect was notable at 0.05 and 1mM concentrations)	↓: CD147, MMP-2, and MMP-9.	NR		2009	[20]
Cytotoxicity	NCI-H460 (inhibition rate was 85.89% at 607.2 μM)	↑: Caspase-3/-9, depolarized cells, ΔΨm disruption, cytoplasm cytochrome c, and Bax/BcL-2 ratio.	NR		2010	[21]
Cytotoxicity	PC-3 and DU-145 (IC_50_ was 121.4 μM for both cell lines)	↓: PGE, COX-2, NF-κB, andp-NF-κB (p65).	NR		2011	[22]
Cytotoxicity	NCI-H460 (607.1 μM)	↓: AKT and ERK1/2.	NR		2012	[23]
Cytotoxicity Invasion and migration inhibition	MDA-MB-231 and 4T1 (SIN displayed cytotoxicity at 1 mM and showed invasion and migration inhibition at 0.25 and 0.5 mM)	↑: CUEDC2. ↓: NF-κB binding to IκB, nuclear translocation of NF-κB, vimentin, tendine-C, CCK, MCP-1, IL-11, NF-κB activation, and p-IKK, IL-4/miR-324-5p.	NR		2015	[24]
Cytotoxicity	A549 (0.25 mM of SIN led to apoptosis)	↑: E-cadherin. ↓: JAK2, STAT3, p-STAT3, Snail, N-cadherin, and vimentin.	NR		2016	[25]
Antitumor (invasion and metastasis inhibition)	HOS and U2OS cells (50–400 µM concentrations were selected for both cell lines)	↑: TIMP-1 and TIMP-2 ↓: CXCR4, p-STAT3, VEGF, CD147, MMP-2, MMP-9, VEGF, RANKL, and p-NF-κB (p65) expression.	150 mg/kg	↓: RANKL-mediated osteolysis, cortical bone destruction, and number of osteoclasts.	2016	[26]
Antitumor	U87 and SF 767 (0.125–0.5 mM concentrations were selected for both cell lines)	↓: Akt-mTOR. ↑: JNK, EB, and lysosome.	75, 150 mg/kg	↑: Cathepsin B/D. ↓: Tumor volume and weight, p62.	2017	[27]
Antitumor	B16-F10 (25–100 mM)	↑: Beclin l, Bax, caspase-3, and LC3II/LC3I ratio. ↓: p-p62/SQSTML, PI3K/Akt/mTOR, and BcL-2.	100 mg/kg	↓: Tumor volume and weight, Ki67, and PCN.	2018	[28]
Antitumor	U87 and U251 (16 mM for both cell lines)	↑: p53 expression. ↓: SIRT1 expression.	100 mg/kg	↓: U87 transplanted tumors growth.	2018	[29]
Antitumor	MDA-MB-231 (0.5 mM)	↓: MMP-2, vimentin, IL-11, NF-κB, and it-mediated Shh pathways.	15 mg/kg	↑: Survival time of mice with lung metastatic breast cancer. ↓: Lung metastasis of breast cancer.	2018	[30]
Cytotoxicity	MDA-MB-231 and MCF-7 (1–16 μM concentrations for both cell lines)	↑: p16, cleaved caspase-3/-9, PDCD-4, and miR-29. ↓: PCNA, Cyclin D1, CDK4, p-JNK, and p-MEK.	NR		2019	[31]
Cytotoxicity	MKN45 and SGC7901 (20 μM)	↑: Bax, cleaved caspase-3, MMP-9, vimentin, AMPK, Wnt/β-catenin, and miR-204. ↓: Cyclin D1 and BcL-2.	NR		2019	[32]
Antitumor	NSCLC (25–100 μM concentrations were selected, but IC_50_ value was not reported)	↓: p-Histone H3 (Ser10), Akt, and downstream kinase S6, HK.	40 mg/kg	↓: Tumor volume and weight.	2020	[33]
Cytotoxicity	Hep3B and HepG2 (2 and 4 mM for both cell lines)	↓: p-AMPK, p-STAT3, and MARCH.	NR		2021	[34]
Cytotoxicity	MDA-MB-231 SP (0.2–1 mM)	↓: N-cadherin, vimentin, and MMP-2, MMP-9, p-PI3K, p-Akt, and p-mTOR.	NR		2021	[35]
Cytotoxicity	HeyA8 (IC_50_ = 1.56 mM)	↓: CDK1, p-CDK (Thr161), and p-Histone H3 (Ser10).	NR		2021	[36]
Antitumor	SK-Hep1 (0.125–1 mM)	↑: Cleaved caspase-9 and cleaved caspase-3. ↓: I3K/AKT1 pathway, PI3K, p85α, AKT1, BcL-2, pro-caspase-9, and pro-caspase-3.	75, 150 mg/kg	↓: Tumor volume and weight.	2022	[38]
Antitumor	HeLa (0.25–1 mM)	↑: Caspase-3. ↓: Cells activity.	70, 140 mg/kg	↑: Tumor cell apoptosis. ↓: Tumor growth, activity of TrxR, and ROS.	2022	[39]
Cytotoxicity	Breast cancer SP cells (0.75 mM)	↑: MiR-340-5P. ↓: SIAH2/HIF-1α pathway and epithelial interstitial transformation.	NR		2022	[40]
Antitumor	HT-29, HCT-116, and SW-480 (2.5 mM for these three cell lines)	↓: IL-1β and TNF-α at mRNA and protein levels; ↑: CPT1A and LPCAT3.	120 mg/kg	↓: Rectal neoplasia production, length of colon, number and volume of tumors, colonic mucosal injury, necrosis, submucosal edema and inflammatory cell infiltration improvement, and colitis-related tumor.	2022	[41]

NR: None reported. ↓: Decrease or inhibition. ↑: Increase or induction. If IC_50_ values were reported, we displayed them in the second column. If IC_50_ values were not reported, we only displayed effective concentrations in the second column.

**Table 2:** Antitumor activity of sinomenine hydrochloride.

Activity	Cell Type or Model In Vitro (Effective Concentrations or IC_50_ Values)	Mechanism of Action	In Vivo		Year	Ref.
			Dose (mg/kg)	Therapeutic Effect		
Antitumor	Hep3B and SMMC7721 (0.5–2 µM for both cell lines)	↑: p21, cytoplasm of Cyt c and Omi/HtrA2. ↓: ∆ψm destruction, BcL-2/Bax ratio, caspase-3/-8/-9/-10, and survivin.	50, 100, 150 mg/kg	↑: Apoptotic cell number. ↓: Tumor weight and volume.	2013	[42]
Antitumor	MDA-MB-231 and MCF-7 (IC_50_ values were 1.33 and 1.51 mM, respectively)	↑: p21, p2, cytochrome c in the cytoplasm, cleaved PARP, Bax/BcL-2 ratios, MAPK activation, p-ERK, p-JNK, p-p38, and ROS. ↓: Cyclin D1, cyclin E, CDK4, MCM7, p-Rb, and ATM/ATR-Chk1/Chk2.	75, 150 mg/kg	↑: Bax/BcL-2 ratio. ↓: Tumor volume and weight, tumor proliferation marker PCNA production.	2014	[43]
Cytotoxicity	ACHN and 786-O (20 μM and 80 μM for both cell lines)	↓: MT and EMT-related transcription factors, MMP 2, MMP 9, nail, and Twist.	NR		2017	[44]
Antitumor	U87 and SF767 (0.0625–0.25 mM for both cell line)	↑: p27, p21, PERK, eIF2α, IRE1α, CCAA, ER stress, and autophagy. ↓: Cyclin D1/D3/E, CDK4, free Ca^2+^, Vimentin, Snail, Slug, NF-κB activation, and MMP-2/-9.	75 mg/kg	↓: Tumor growth.	2018	[45]
Cytotoxicity	MDA-MB-231 (15.2 mM)	↓: Cells growth and bacterial growth.	NR		2018	[46]
Antitumor	HeLa (1 mM)	↑: DNA damage, Chk1 activity, and cell cycle checkpoint. ↓: DDR factors KU80 and RAD51 expression.	100 mg/kg	↓: Tumor growth.	2018	[47]
Antitumor	Eca109, EC9706 (IC_50_ values were 0.3 and 0.4 mM for Eca109 and EC9706 cell lines, respectively)	↑: Bax. ↓: BcL-2, cyclin B1, CDK1, Ku86, Ku70, and Rad5.	75 mg/kg	↓: Tumor growth.	2018	[48]
Cytotoxicity	SK-Hep 1 (0.25 m)	↑: CXCL12, CXCR4, CCR7, and CCL21. ↓: ERK1/2/MMP-2/-9 signaling pathway.	NR		2022	[49]
Cytotoxicity	BCPAP and PTC-1 (4 mM concentration was selected for both cell lines)	↑: Thyroid iodine-processing genes, NIS, TSHR/cAMP signaling pathway, and RAI uptake. ↓: PTC cell proliferation.	NR		2022	[50]
Antitumor	H1819 (50 µM) and H1975 (200 µM)	↑: p-AMPK. ↓: p-mTOR.	25, 50, 100 mg/kg	Comparable to that of cisplatin group, but toxicity was lower.	2023	[51]

NR: None reported. ↓: Decrease or inhibition. ↑: Increase or induction.

**Table 3:** Combination strategies for synergetic enhancement between SIN and other drugs.

Combined Drugs	Cell Type or Model In Vitro (Effective Concentrations or IC_50_ Values)	Mechanism of Action	In Vivo		Year	Ref.
			Dose (mg/kg)	Therapeutic Effect		
Aclarubicin	HL-60 (15.2–60.7 Nm of SIN)	↑: Caspases-3/-9. ↓: PGE, PGE2, COX-2, and NF-κ.	NR		2011	[52]
5-FU	MKN-28, SGC-709, BGC-823 and HGC-27 (20–80 µM of SIN for these four cell lines)	↑: Transfer of cytochrome c from mitochondria to cytoplasm, caspase-3/-9. ↓: TS mRNA levels.	10 mg/kg	↓: Tumor volume and weight in combination group.	2013	[53]
5-FU	HepG2 (3.9 mM of SIN combined with 44.92 mM of 5-FU)	↓: Cell activity.	NR		2021	[54]
Adriamycin	Caco-2 and MDR-Caco-2 (500 mM of SIN for both cell lines)	↓: PGE2, P-gp/MDR1, COX-2, and NF-κB.	NR		2014	[56]
Cisplatin	A549 (50 μM of SIN combined with 3372.5 mM of Cisplatin)	↑: miR-200a-3p. ↓: Glutamine metabolism.	NR		2022	[57]
Tacrolimus and mycophenolic acid	PBMC (10–1000 μM of SIN)	↓: Thymidine incorporation, interleukin-2 synthesis, and T lymphocyte cell cycle progression.	NR		1999	[58]
MTX	RA-FLS (303.6 μM of SIN)	↑: OPG and ratio of OPG/RANKL. ↓: RANKL, OPN, IL-6, IL-17, MMP-1, and MMP-3/-13.	120 mg/kg	↓: Synovial inflammation and joint injury.	2014	[59]

NR: None reported. ↓: Decrease or inhibition. ↑: Increase or induction. If IC_50_ values were reported, we displayed them in the second column. If IC_50_ values were not reported, we only displayed effective concentrations in the second column.

**Table 4:** Anti-inflammatory activity and analgesic activity of SIN.

Activity	Cell Type or Model In Vitro (Effective Concentrations or IC_50_ Values)	Mechanism of Action	In Vivo		Year	Ref.
			Dose (mg/kg)	Therapeutic Effect		
Anti-inflammatory	NR		150 mg/kg	Joint swelling and ESR.	1996	[71]
Anti-inflammatory	PMs and synoviocytes (91.1–364.3 μM for both cell lines)	↑: IκBα. ↓: TNF-α, IL-1β, and NF-κB.	NR		2005	[72]
Anti-angiogenic	HUVEC (125–1000 μM)	NR	NR		2005	[73]
Anti-inflammatory	PMs and synoviocytes (276.5–1105.9 μM for both cell lines)	↑: IκBα. ↓: TNF-α, IL-1β, and NF-κB.	NR		2005	[74]
Anti-colitis	NR	↓: TNF-α and IFN-γ.	100, 200 mg/kg	↑: Myeloperoxidase activity. ↓: Body weight, macroscopic score, and histological score.	2007	[77]
Inhibited activation of retinal microglia cells	Retinal microglia cells (0.1 mM and 1 mM y)	↓: TNF-α, IL-1β, IL-6, ROS, and nuclear translocation of NF-κB p65.	NR		2007	[78]
Analgesic	CHO cells (1 μM and 10 μM)	↑: p-OMR.	10, 20, 30 mg/kg	↑: OMR activation.	2008	[79]
Anti-OA	NR	↑: TIMP-1/-3. ↓: IL-1β, IL-6, and MMP-2/-9.	100 mg/kg	↓: Incidence and progression of CIA, foot swelling, ESR, and arthritis score.	2008	[80]
Anti-MSPGN	NR	↓: T-bet, T-bet/GATA-3 ratio, and IFN-γ.	240 mg/d	↓: Albuminuria. ↑: Complement C3.	2009	[81]
Anti-OA	SW1353 and human osteoarthritic chondrocytes (1–5 mM for both cell lines)	↓: MMP-1, MMP-3, MMP-9, and MMP-13, catabolism of IL-1β, and proteolytic enzymes.	NR		2010	[82]
Anti-OA	Chondrocytes (10–250 mM)	↑: TIMP-1. ↓: IL-1, β-induced GAG, and MMP-13.	NR		2010	[83]
Anti-inflammatory	FLS and THP-1 (0.01–1.00 mM for both cell lines)	↓: Invasion and migration ability, CD147, and MMP-2/-9.	NR		2011	[84]
Anti-inflammatory	RA-FLS (75.9–607.2 μM)	↓: VCAM-1, IL-6, CCL 2, CXCL8, p-IκBα, and NF-κB.	NR		2011	[85]
Anti-inflammatory	HMC-1 (IC_50_ = 52.73 μM)	↓: TNF-α, IL-6, IL-8, COX-2, p-ERK1/2, p-p38 MAPK, p-κBα, and NF-κB.	NR		2012	[86]
Anti-RA	RAW264.7 (0.0625–1 mM)	↓: *c-Src*, *MMP-9*, *TRACP*, TRAF6, NF-κB, IκBα degradation and translocation of p65 to the nucleus, p-p38 and p-JNK, Ca^2+^ influx, NFATc1, AP-1, *Fra-1*, *Fra-2*, and c-Fos.	80 mg/kg	↑: Body weight. ↓: Hind paw swelling and bone loss.	2013	[87]
Anti-colitis	NR	↓: MPO activity, miR-155, c-Maf, TNF-α, and IFN-c.	100, 200 mg/kg)	↑: Weight and survival rate, colon symptoms, and histological scores. ↓: Diarrhea score.	2013	[88]
Anti-RA	NR	↓: TNF-α, IL-1β, and IL-6.	100 mg/kg	↓: Synovial hypertrophy, cartilage damage, joint space narrowing, osteoporosis, cartilage, and bone erosion.	2013	[89]
Analgesic	NR	GABA_A_	10–40 mg/kg	↑: Paw withdrawal threshold. ↓: Duration of immobile behavior, depression-like behavior, and chronic pain.	2014	[90]
Anti-macrophage activation	RAW264.7 (100 μM)	↓: TNF-α, IL-6, α7nAChR, and NF-κB p65. ↑: Cytoplasmic IκBα.	NR		2015	[91]
Anti-arthritic	RA-FLS (0.125–1 mM)	↓: ALP activity, MyD88, and TRAF-6.	NR		2015	[92]
Analgesic	NR	GABA_A_.	5–80 mg/kg	Analgesic effect on postoperative rats via GABA_A_ receptor.	2016	[93]
Anti-inflammatory	NR	TGF-β1/CTGF pathway and oxidative stress.	25, 50, 75 mg/kg	Asthmatic mice airway inflammation and remodeling alleviation	2016	[94]
Anti-arthritic	Treg cells (0.1–1 mM)	↑: IL-10 level. ↓: Foxp3, IL-10, RoRγT, IL-17a, IL-17f, IL-21levels, Th17 cells, Treg cells, L–1β, TNF-α, IL-6, and IL-17.	120 mg/kg	↓: Arthritis index, inflammation and cartilage damage, and paw swelling.	2016	[95]
Anti-neuropathic pain	HEK293 (10 μM)	↓: ATP activation, P2X3, p-P38MAPK, and pain behavior alleviation.	40 mg/kg	MWT (about 50%) and TWL (about 80%) enhancement	2017	[96]
Anti-inflammatory bowel disease	NR	↑: SIGIRR and IL-10. ↓: TLR/NF-κB, IFN-γ, IL-1β, TNF-α, IL-12p70, and IL-6.	30, 90, 270 mg/kg of SIN; 180, 540, 1600 mg/kg of SIN microspheres	The colon length of SIN microspheres group was longer than SIN group; histological grade score of SIN microspheres group was lower than SIN group.	2017	[97]
Anti-endotoxin	Endothelial cells (3 mM)	↓: Key control genes in the pathogenesis of LPS.	NR		2018	[98]
Anti-microglial inflammatory response	BV-2 (25–100 μM)	↑: IκB-α and miRNA-183-5p. ↓: SP1/miRNA-183-5p/IκB-α pathway, p-p65, p-p50, TNF-α, IL-1β, IL-6, and SP1.	NR		2018	[99]
Anti-RA	RAW264.7 (3–151.8 µM)	↓: IL-6, GM-CSF, IL-12p40, IL-1α, TNF-α, IL-1β, KC (CXCL1), Eotaxin-2, IL-10, M-CSF, RANTES, and MCP-1.	50, 100 mg/kg	↓: Swollen paw score, inflammation score, and cartilage damage score of CIA mice, weight loss.	2018	[100]
Anti-arthritis	PC12 (0.03–0.3 mM)	↑: α7nAChR-PI3K/Akt/mTO.	120 mg/kg	VIP production promotion in the gut and neuronal cells.	2018	[101]
Anti-inflammatory pain	NR	↓: P38MAPK, NF-κB, TNF-α, IL-1b, IL-6, p-p65, p-p3, COX-2, and PGE2.	30 mg/kg	↓: Inflammatory pain.	2018	[102]
Anti-inflammation of eye tissue	NR	↓: NF-κB, TNF-α, PG-E2, and translocation of NF-κB p65 subunits to the nucleus.	50, 100 mg/kg	↓: Number of inflammatory cells, protein leakage.	2018	[103]
Anti-arthritis	NR	↑: OD and MDA. ↓: NF-κB and MAPK, TNF-α, IL-6, IL-1β, IL-8, COX-2, iNOS, and MMP-2/-9.	50 mg/kg	↑: Total body weight of the rat. ↓: Paw volume and arthritis score.	2018	[104]
Anti-colitis	NR	↑: Nrf2/NQO-1 and SOD activity. ↓: TNF-α, IL-6, and iNOS level.	100 mg/kg	↓: Body weight and DAI score, colon shortening, and colitis histological damage.	2018	[105]
Anti-inflammatory	MG63 (0.25–1 mM)	↑: SOD, CAT, Nrf2, HO-1, NQO-1, and Nrf2. ↓: MAPKP38/NF-κB, IL-1β, IL-6, TNF-α, p-p38, p-NF-κB (P65), and MDA.	NR		2018	[106]
Analgesic	NR	↓: JAK2/STAT3 and CAMKII/CREB.	10, 20, 40 mg/kg	Mechanical hypersensitivity of pain in cancer bone algia rat alleviation, and microglia activation inhibition.	2018	[107]
Anti-RA	NR	↑: IL-10. ↓: IL-1β, IL-6, and TNF-α.	1.5 g/kg	The extract (4:1, 1.5 g/kg) and extract (3:1, 1.5 g/kg) groups were superior to SIN.	2018	[108]
Anti-OA	Mouse chondrocytes (6.25–25 μM)	↑: Nrf2/HO-1. ↓: NF-κB, iNOS, COX-2, NO, PGE2, TNF-α, IL-6, P-NF-κB p65, p-IκBα, ADAMTS-5, and MMP.	10 mg/kg	↑: Thickness of articular cartilage. ↓: Degradation of ECM.	2019	[109]
Anti-OA	Mouse chondrocyte (30 μM)	↓: IL-6, TNF-α, MiR-192, NF-κB, and MAPK.	NR		2019	[110]
Anti-RA	NR	↓: TNF-α, IL-6, ROS, and ESR.	5 mg/kg (AS-TE)	↓: Joint swelling, bone defects.	2019	[111]
Anti-inflammatory	HaCaT (1 μM)	↓: IL-6, TNF-α, COX-2, iNOS, p-P65, p-IκBα, p-p38-MAPK, NF-κB, MAPK, and CAT1.	NR		2019	[112]
Anti- inflammatory	NR	↓: α7nAChR.	120 mg/kg	↓: Paw swelling, AI, TNF-α, and ESR.	2019	[113]
Anti-inflammatory	Raw264.7 (300 μM)	↑: p-STAT3 and JAK2/STAT3. ↓: TNF-α, MCP-1, MIF, MMP-9, CD14, TLR4, intracellular Ca^2+^, and NF-κB.	NR		2019	[114]
Analgesic	NR		0–80 mg/kg	Analgesic effect of older pups was better than that of younger pups.	2020	[115]
Anti-inflammatory	Macrophage (303.6–3036 μM)	↓: TNF-α, IL-1β, IL-6, TLR4, MyD 88, p-IκB, macrophage immune response, and TLR4/NF-κB.	NR		2020	[116]
Anti-inflammatory	Raw264.7 (3–75.9 μM)	↑: SOCS1. ↓: TNF-α, IL-1β, IL-6, inflammatory responses, miR-155, and NF-κB.	NR		2020	[117]
Anti-rheumatoid arthritis	NR		2 mg/kg	Target RA site. The leakage of SIN prevention.	2020	[118]
Promote MMP production	SW1353 (25–100 µM)	↑: SOCS3. ↓: MMP, p-JAK2, and p-STAT3.	NR		2020	[119]
Anti-inflammatory	Raw264.7 (10–200 μM)	↑: IL-6, TNF-α, and NF-κB nuclear translocation. ↓: ROS and LDH.	NR		2020	[120]
Anti-inflammation of dorsal root ganglion	DRG (800 μM)	↓: P38MAPK, CREB, c-fos, p-CAMKII, NF-κB, COX2, TLR4, IL-1B, IL-17A, and p38MAPK/CREB.	20 mg/kg	↓: MWT and TWL.	2021	[121]
Anti-RA	PBMC (0.3–30 μM)	No direct effect on T cells.	NR		2021	[122]
Anti-foodborne enteritis of fish	NR	↓: TNF-α, IL-10, IL-22, and FOXP3a.	35 ppm	↑: Intestinal villus height ↓: Inflammation and dysregulation.	2021	[123]
Anti-neuropathic pain	NR	↓: NF-α, IL-1β, IL-6; RIP3, p-JNK, c-Fos, and IP3/JNK.	20, 40mg/kg	↑: Survival neurons of spinal dorsal horn.	2021	[124]
Anti-migration and anti-inflammatory	RAW264.7 and BMDMs (160–640 μM for both cell lines)	↓: RC/FAK/P130CAS, iNOS/NO, integrin αV, integrin β3, TNF-α, and IL-6.	25, 50, 100 mg/kg	↓: Migration of Mouse mononuclear macrophage leukemia cells Raw264.7 to the foot and swelling of the foot.	2021	[125]
Anti-arthritic	RASFs (12.5–100 μM)	↑: Degradation of Keap1, HO-1, and p- p62 (Thr269/Ser272). ↓: IL-6, IL-33, ROS, Nrf2, and p-p62 (Ser35).	25, 50, 100 mg/kg	↓: Incidence rate of CIA mice and swelling of the hind paws.	2021	[126]
Anti-RA	FLSs (200 μM)	↑: A_2A_R and cAMP. ↓: MCP-1, IL-6, vascular endothelial growth factor, and NF-κB pathway.	120 mg/kg	↓: Arthritis index, the hind paw volume, ESR, and TNF-α.	2021	[127]
Anti-RA	NR	↑: Solubility. ↓: Drugs release.	NR		2021	[10]
Anti-colitis	NR	↑: IL-10 and arginine 1. ↓: TNF-α, IL-6, inducable nitric oxide synthase, NOD-, LRR-, and NLRP3 inflammasome.	100 mg/kg	Intestinal microbial composition alteration.	2021	[129]
Anti-pneumonia	WI-38 (5–20 μM)	↓: TNF-α, IL-1β, MCP-1; IL-10, and GSTM 1.	NR		2022	[130]
Anti-arthritis	RAW264.7 cell (50–400 µM)	↓: TNF-α and inflammatory factors.	25, 50, 100 mg/kg	↓: Mean joint score and foot volume.	2023	[51]

NR: None reported. ↓: Decrease or inhibition. ↑: Increase or induction. If IC_50_ values were reported, we displayed them in the second column. If IC_50_ values were not reported, we only displayed effective concentrations in the second column.

**Table 5:** Neuro-related diseases and psychiatric disorders treatment by SIN.

Activity	Cell Type or Model In Vitro (Effective Concentrations or IC_50_ Values)	Mechanism of Action	In Vivo		Year	Ref.
			Dose (mg/kg)	Therapeutic Effect		
Anti-PD	Microglia and mesencephalic glial cells. (10^−14^ M for both cell lines)	↓: ROS, NO, iNO, TNF-α, PGE2, COX-2, and microglial superoxide production.	NR		2007	[151]
Neuroprotective	BV2, HT22 and primary hippocampal cells (0.4 mM for both cell lines)	↓: ROS, NO, TNF-α, IL-6, and MCP-1.	NR		2011	[152]
Anti-TBI	NR	↑: BcL-2, ARE pathways, Nrf2, and GP_X_ and SOD activities. ↓: Caspase-3 and MDA.	10, 30, 50 mg/kg	↑: Motor ability recovery promotion ↓: Brain edema reduction.	2016	[153]
Anti-ischemic stroke	Primary mixed glial cell (0.1–1.0 mM)	↓: NLRP3, ASC, caspase-1, IL-1β, IL-6, IL-18, TNF-α, and OGD-induced NLRP3 inflammasome activation.	10, 20 mg/kg	↓: Cerebellar infarct size, brain water content, neuronal loss, and neurological deficit.	2016	[154]
Anti-ICH	Microglia cell (1 mM)	↑: M2. ↓: Microglia migration, M1, and microglia-mediated neuronal toxicity.	100 mg/kg	↓: Infiltration of microglia activation. ↑: Rain water content and nerve damage and microglia M2 polarization.	2016	[155]
Hypnosis	NR	↑: Flow of Cl^−^ in hypothalamic neurons, glutamic acid decarboxylase (GAD 65/67), and hypothalamic GABA subunits (α4, β1, β2, γ3).	20, 40 mg/kg	↓: Spontaneous activity inhibition and sleep latency of pentobarbital. ↑: Total sleep time.	2017	[156]
Prevention of morphine-induced CPP	SH-SY5Y cells (100 µM)	↓: NMDAR 1/CAMKII/CREB, cAMP and Ca^2+^, p-NMDAR1/NMDAR1, p-CAMKII/CAMKII, and p-CREB/CREB.	60 mg/kg	↓: Astrocyte activation.	2018	[157]
Anti-epileptic	NR	↓: NLRP 1 inflammasome complex, IL-1β, IL-18, IL-6, and TNF-α.	20, 40, 80 mg/kg	↑: Neuroprotective effects: kindling acquisition process disruption and seizure latency. ↓: Seizure duration, spatial learning, and memory damage.	2018	[158]
SIN reduced formalin-induced injurious behavior in mice	NR	↓: p-ERK1/2.	80 mg/kg	↓: Formalin-induced licking and biting responses.	2019	[159]
Anti-SCI	PC12 (10 μM)	↑: Nuclear Nrf2, and Nrf2 nuclear translocation. ↓: IL-1β, IL-6, and TNF-α inhibition.	40 mg/kg	↓: Spinal cord edema.	2019	[160]
Anti-AD	C8D1A (100 μM)	↓: ROS, NO and IL-12p70, IL-10, IL-6, IL-1β, and IL-8, and toxic factors.	NR		2020	[161]
Effects of SIN on orphine-induced zebrafish	NR		40, 80 mg/kg	↑: TH and NR2B, zfmor, zfdor1, and zfdor2. ↓: CPP effect.	2021	[162]
Anti-MS	NR	↑: IL-10. ↓: IL-1β, IL-6, IL-18, TNF-α, and IL-17A.	100 mg/kg	Neuroinflammation, demyelination, axon damage, and loss alleviation.	2021	[163]
Anti-brain injury	BV2 (50–200 µM)	↑: M2 markers (Arg 1 and IL 10), Nrf2, HO1, and NQO1 ↓: TNF-α, IL-1β, NOS2, SOD, GPx, M1 markers (NOS2 and IL 6), p-IκBα, and nuclear translocation of NF-κB.	20 mg/kg	↓: Pathological lesion of brain tissue and water content of brain.	2021	[164]
Cognitive dysfunction promotion	NR		100 mg/kg	↑: Short-term Y-maze alternations, dark avoidance latency of passive avoidance pattern. ↓: Detection error and latency of Barnes maze task and cognitive dysfunction.	2022	[165]
Anti-PD	NR	↑: Beclin 1, LC3-II/LC3-I ratio, and LC3B-positive neurons ↓: PI3K/Akt/mTOR pathway and P62.	20 mg/kg	Motor function of PD mice improvement and survival of dopaminergic neurons promotion.	2022	[167]
Anti-IVDD	RAW264.8 and NPCs (75.9 μg/mL for both cell lines)	↑: ARG-1, M1 to M2, IL-10, type II collagen, SOD, and BcL-2. ↓: iNOS, TNF-α and IL-6, ROS and MDA, Bax, caspase-3, MMP-2, and MMP-9.	NR		2022	[166]
Diabetic peripheral neuropathic pain (DPNP) alleviation	NR	↓: PTGS2.	1 mL/kg	↓: Blood glucose levels and increased body weight.	2023	[168]
SAH-induced early brain injury (EBI) remission	NR	↑: BcL-2. ↓: Bax, IL-1β, IL-6, and SAH-derived microglia.	50, 100 mg/kg	↓: Water content of cerebral cortex and apoptotic fraction.	2023	[169]

NR: None reported. ↓: Decrease or inhibition. ↑: Increase or induction. If IC_50_ values were reported, we displayed them in the second column. If IC_50_ values were not reported, we only displayed effective concentrations in the second column.

**Table 6:** Immunosuppression activity of SIN.

Activity	Cell Type or Model In Vitro (Effective Concentrations or IC_50_ Values)	Mechanism of Action	In Vivo		Year	Ref.
			Dose (mg/kg)	Therapeutic Effect		
Immunosuppression	NR		30, 100 mg/kg	↓: Anti-SRBC PFC, number of spleen cells.	1985	[170]
Kidney protection	TECs CD4^+^ T (303.6 μM)	↑: IL-2 and IFN-γ. ↓: B7-H1 and B7-DC.	NR		2005	[171]
Inducing CD4T cell apoptosis	CD4^+^ T (0.1 mM and 1 mM)	G1 phase blockade ↓: Caspase-3 cleavage.	NR		2007	[172]
Anti-MS	NR		50, 100, 200 mg/kg	↓: EAE clinical scores, percentage of initial weight loss, TNF-α, IFN-g, MIP-1A, and MCP-1.	2007	[173]
Promoting DC differentiation	DC (607.2 μM)	↑: Differentiation of monocytes into DCS, CD1a. ↓: CD14, CD86, CD40, B7-H1, HLADR, CD32, MR reversion, IFN-γ, and IL-2 production.	NR		2007	[174]
Anti-rheumatic heart disease	HUVECs (0.5 M and 1.0 M)	↓: VCAM-1.	NR		2007	[175]
Promoting DC maturation	DC (2 mM and 5 mM)	↓: HLA-DR, CD40, CD80, CD86, CD83, CPM value, IL-1, NF-κB, p-IκBα, and migration of RelB from cytoplasm to nucleus.	NR		2007	[176]
Promoting RBL-2H3 activation	RBL-2H3 (0.5–2 mM)	↓: β-amino-hexosamine release, IL-4, TNF-α, p-GAB2, p-Akt, and p-p38MAPK.	NR		2008	[177]
Inhibiting L-histidine decarboxylase	L-histidine decarboxylase (IC_50_ = 969 mM, KI = 762 mM)	↓: L-histidine decarboxylase inhibition.	NR		2009	[178]
Promote cell threshing	RBL-2H3 (31.25–2 mM)	↑: β-hhexosaminidase, P-CPLA2, p-ERK promotion, ANXA1 cleavage, and COX-2. ↓: Degranulation of RBL-2H3 cell induction.	NR		2015	[179]
Anti-macrophage activation	P815 (0.1–100 μM)	↑: β-hexosaminidase release, IP3, intracellular Ca^2+^, IP 3R, p-Lyn, and PLCγ1. ↓: Histamine release.	0.364, 1.82, 9.10 mg/kg	↑: Mouse ear vascular permeability, IP3, and TNF-α.	2016	[180]

NR: None reported. ↓: Decrease or inhibition. ↑: Increase or induction. If IC_50_ values were reported, we displayed them in the second column. If IC_50_ values were not reported, we only displayed effective concentrations in the second column.

**Table 7:** Anti-depression activity of SIN.

Activity	Cell Type or Model In Vitro (Effective Concentrations or IC_50_ Values)	Mechanism of Action	In Vivo		Year	Ref.
			Dose (mg/kg)	Therapeutic Effect		
Anti-depression	NR	↑: BDNF, pTrkB, and pCREB.	20, 40 mg/kg	↑: Social interaction and sucrose preference. ↓: Forced swimming and tail suspension tests immobility.	2018	[182]
Anti-depression	NR	↑: NE and 5-HT. ↓: Imbalance of hippocampal neurotransmitter, IL-1b, IL-6, TNF-α, P38-MAPK-NF-κB, NLRP 3, ASC, and caspase-1.	30, 100, 300 mg/kg	↑: Weight and sucrose consumption. ↓: Depressive symptoms alleviation, time of forced swimming, and tail suspension tests.	2018	[183]

NR: None reported. ↓: Decrease or inhibition. ↑: Increase or induction. If IC_50_ values were reported, we displayed them in the second column. If IC_50_ values were not reported, we only displayed effective concentrations in the second column.

**Table 8:** Anti-sepsis activity of SIN.

Activity	Cell Type or Model In Vitro (Effective Concentrations or IC_50_ Values)	Mechanism of Action	In Vivo		Year	Ref.
			Dose (mg/kg)	Therapeutic Effect		
Anti-sepsis	PM (100 μM)	↑: Autophagosome formation. ↓: IL-6 and TNF-α release.	100 mg/kg	CLP-induced organ damage attenuation.	2015	[184]
Anti-ALI	RAW264.7 (25–100 μM)	↑: Nrf2 and autophagy pathways, Nrf2, HO-1, SOD, LC-3II, ATG5, and Beclin1. ↓: KEAP1, NQO1, IL-6, TNF-α, and serum content of MD.	100 mg/kg	↓: LPS-induced lung injury and W/D ratio of lung tissue.	2020	[185]
Anti-septic lung injury	Caco-2 (50–400 μM)	↑: Aromatic hydrocarbon receptor, CYP1A1, Claudin1, Nrf2, HO-1, and NQO-1.	100 mg/kg	Intestinal microbiome regulation, intestinal barrier restoration, composition of beneficial and harmful bacteria changed, endotoxins produced prevention, inflammatory response inhibition, and acute lung injury protection.	2021	[186]

NR: None reported. ↓: Decrease or inhibition. ↑: Increase or induction. If IC_50_ values were reported, we displayed them in the second column. If IC_50_ values were not reported, we only displayed effective concentrations in the second column.

**Table 9:** Kidney protection of SIN.

Activity	Cell Type or Model In Vitro (Effective Concentrations or IC_50_ Values)	Mechanism of Action	In Vivo		Year	Ref.
			Dose (mg/kg)	Therapeutic Effect		
Anti-kidney injury	NR	↑: Nephrin and podocin, PPAR-α. ↓: TNF-α and IL-1β.	10, 30 mg/kg	↑: Total protein and serum albumin ↓: Weight loss, foot process width of rats, and urinary protein excretion cholesterol and triglycerides.	2012	[187]
Anti-kidney injury	NR	↓: XCL-10, ICAM-1, TNF-α, IL-6, p-IKK-β, IκB-α, NF-κB, MAPk, p-P44/42, p-JNK, and p-P38.	200 mg/kg	↓: Serum CR and BUN, renal histological damage, and inflammatory infiltration.	2013	[188]
Anti-kidney injury	HK-2 (0.1–50 μM)	↑: MiR-124. ↓: Apoptosis.	200 mg/kg	↑: SOD. ↓: MDA, MPO, inflammatory infiltration, and renal cell apoptosis induced via I/R.	2016	[189]
Anti-Kidney Injury	HEK293 (25–100 μM)	↑: Nrf2, HO-1, NQO1, ARG-1, and IL-10. ↓: Keap1.	100 mg/kg	↓: Renal fibrosis, E-Cadherin, α-SMA and fibronectin, IL-1β, and TNF-α.	2016	[190]
Anti-renal fibrosis	HEK293 (25–100 μM)	↑: Nrf2, HO-1, NQO1, and NRF2. ↓: GFβ/Smad Wnt/β-catenin, and TGFβ-induced ROS levels.	100 mg/kg	↓: Renal tubular dystrophy and fibrosis area.	2016	[191]
Anti-diabetic nephropathy (DN)	HrGECs induced by hyperglycemia (HG) (50 μM and 100 μM)	↑: Activation of C/EBP-α/Claudin-5 axis. ↓: Production of IL-18 and IL-1β within cells.	20, 40 mg/kg	↓: Blood glucose, body weight, kidney tissue pathology, infiltrating inflammatory cell, and alleviated glomerular endothelial dysfunction.	2022	[192]
Anti-renal fibrosis	NR	↑: PIK3CB TGF-β1, miR-204-5P, autophagy induction, and M2-type polarization. ↓: Creatinine and BUN, PI3K/Akt, pro-inflammatory cytokine, and M1-type polarization.	50, 100 mg/kg	↓: Body weight and kidney weight.	2023	[193]
Anti-cisplatin (CP)-induced kidney injury	HK2 (IC_50_ = 280 μM)	↑: p21, Bax, Noxa, PARP1, BcL-2, and SIRT6. ↓: HO-1, 4-HNE, 3-NT, TNF-α, STAT3, p-STAT3, NF-κB p65, and caspase-3/-8.	5 mg/kg	↓: Blood BUN, creatinin, NGAL, KIM-1, and histopathological scores.	2023	[194]

NR: None reported. ↓: Decrease or inhibition. ↑: Increase or induction. If IC_50_ values were reported we displayed them in the second column. If IC_50_ values were not reported, we only displayed effective concentrations in the second column.

**Table 10:** Osseous tissue and brain protection of SIN.

Activity	Cell Type or Model In Vitro (Effective Concentrations or IC_50_ Values)	Mechanism of Action	In Vivo		Year	Ref.
			Dose (mg/kg)	Therapeutic Effect		
Promoting osteoclast apoptosis	RAW 264.7 (0.25–2 mM)	Activating caspase-3.	NR		2014	[195]
Anti- lipopolysaccharide-induced osteoclastogenesis and osteolysis	RAW264.7 cell (0.25–1 mM)	↓: Tracp, MMP-9, C-src, integrin αVβ3 and CK, TNF-α production, c-fos, Fra-1 and Fra-2, intracellular Ca^2+^ influx, TLR4, TRAF6, MAPK (p-P38), NF-κB, AP-1, NF-ATc1, and TLR.	25, 50, 100 mg/kg	↓: Craniolysis and TNF-α.	2016	[196]
Regulation of MSCs	MSCs (0.25 mM)	↑: OPG. ↓: PTGES3, PGE2, and RANKL OPG/RANKL ratio.	NR		2017	[197]
Anti-brain injury	BV-2 microglia (0.1 mM and 1 mM)	↓: Erythrocytolysis-induced TNF-α, IL-1, IL-6, ROS, ICH induced microglial activation, NF-B, and migration.	20 mg/kg	↓: Brain water content and neurological deficit score.	2014	[198]
Anti-ischemic	Astrocyte (1 mM)	↑: CRYAB/STAT3, DRD2, CRYAB, CRYAB, and CRYAB interaction with STAT3. ↓: STAT3, p-STAT, CRYAB nuclear translocation, and STAT3 DNA-binding activity.	10, 20 mg/kg	↓: Ischemic infarct volume and neuronal apoptosis, neurological impairment.	2016	[9]
Anti-TBI	RAW264.8 (151.8–910.8 μM)	↓: Early/acute inflammatory responses, TNF-α, IL-1β, CCL-3, IL-6, Oxidative stress (iNOS and NO), and NF-κB and its nuclear shift.	30 mg/kg	Specifically targets activated microglia/macrophages.	2020	[199]

NR: None reported. ↓: Decrease or inhibition. ↑: Increase or induction. If IC_50_ values were reported, we displayed them in the second column. If IC_50_ values were not reported, we only displayed effective concentrations in the second column.

**Table 11:** Cardiovascular tissue protection of SIN.

Activity	Cell Type or Model In Vitro (Effective Concentrations or IC_50_ Values)	Mechanism of Action	In Vivo		Year	Ref.
			Dose (mg/kg)	Therapeutic Effect		
Anti-vascular injury	VSMC (200 μM)	↓: Phosphorylation of ERK1/2 and p38, Akt, GSK3β, STAT3, and PDGFR-β.	150 mg/kg	↓: Formation of neointima and number of PCNAP cells.	2013	[200]
Improving renal function	HRGECs (25–100 mM)	↑: Occludin, Nr. ↓: RhoA/ROCK signal transduction activation, abnormal occlusive protein distribution reversion, RhoA/ROCK, Cell permeability, and ROS.	NR		2016	[201]
Improving cardiac function	NR	↓: NF-κB and cytokines, IκB expression, CD3^+^- and CD68^+^-positive cells infiltration, TNF-α, IL-1, and IL-6 level.	30, 60, 120 mg/kg	↑: Heart rate and EF. ↓: Cardiac function and pathological symptoms, IVSD, LVEDD, LVESD, cardiac index, and cardiomyocyte hypertrophy.	2017	[202]
Anti-CH	H9C2 cells (50–100 μM)	↓: Cell surface area and apoptosis rate, ROS and MDA, Caspase-3, and Bax. ↑: BcL-2 and Nrf2/AR.	40, 80 mg/kg	↓: Heart weight and left-ventricular mass index.	2021	[203]
Anti-CH	NR	↓: NF-κB, TNF-α, and IL-1β.	120 mg/kg	↑: Level of SOD. ↓: LVWI, LVAWd and LVPWd, degree of myocardial hypertrophy, inflammatory cell infiltration, interstitial fibrosis, and contents of LDH and MDA.	2021	[204]
Anti-I/R	NR	↑: SOD, GPx and CAT. ↓: CK-MB, CK, Tnl, TXB 2, TF, Fbg, PA1-1, MDA, LDH, AST, Hs-CRP, MCP-1, NF-α, IL-1β, and IL-6.	5, 10, 20 mg/kg	↓: Frequency, duration, and incidence of VT and VF, incidence of VEB, and area of myocardial infarction.	2022	[205]

NR: None reported. ↓: Decrease or inhibition. ↑: Increase or induction. If IC_50_ values were reported, we displayed them in the second column. If IC_50_ values were not reported, we only displayed effective concentrations in the second column.

**Table 12:** Liver protection and respiratory protection of SIN.

Activity	Cell Type or Model In Vitro (Effective Concentrations or IC_50_ Values)	Mechanism of Action	In Vivo		Year	Ref.
			Dose (mg/kg)	Therapeutic Effect		
Anti-inflammatory	NR		10–100 mg/kg	↓: Liver injury, TNF, and ROS.	1994	[206]
Anti-acute liver injury	BRL-3A (30.4–303.6 μM)	↑: SOD and GSH-Px. ↓: TGF-β/Smad, MDA, LDH d, TNF-α, IL-1β, IL-6, NLRP3, ASC, caspase-1, IL-1β, and NLRP3.	100 mg/kg	↓: Liver dysfunction.	2020	[207]
Anti-IR in liver	NR	↑: TNF-α, IL-6, IL-8, IL-10, and Nrf-2/HO-1.	100 mg/kg	↓: ALT, AST, LDH, inflammatory cell infiltration, and proportion of cytoplasmic vacuoles and necrotic cells.	2022	[208]
Protect liver injury caused by Pb	NR	↑: BcL-2. ↓: Caspase-3, Bax, IL-1β, TNF-α, NF-κB, NF-κB p65, and IκBα.	100 mg/kg	↑: T-AOC, body weight of Pb mice. ↓: Liver index, serum ALT, AST, LDH, liver lead content of Pb mice, and MDA level.	2023	[209]
Treatment of acute lung injury	NR	↑: SOD, NQO1, Nrf2, HO-1, PKC, and Nrf2. ↓: Nrf2/Keap1/PKC, p-NF-κB p65, cytokines, NF-κB, TNF-α, IL-6, IL-1β, MDA, and Keap1.	100 mg/kg	↓: Lung W/D ratio, neutrophil infiltration, pulmonary edema, alveolar wall thickening and cell structure destruction, level of BALF, BALF neutrophils, and MPO.	2018	[210]
Anti-airway remodeling	16Hbe (607.2 mM)	↓: Cell migration, MMP7, MMP9, and vimentin.	35, 75 mg/kg	↓: IgE, IL-4, airway remodeling alleviation, subepithelial collagen deposition, EMT, TGF-B1, and Smad3.	2021	[211]
Anti-cough	NR	↓: TRPVl, SOX5, intracellular Ca^2+^, SP, and NKA.	5000 mg/kg	↓: Capsaicin-induced high cough sensitivity and inflammatory cell infiltration.	2021	[212]

NR: None reported. ↓: Decrease or inhibition. ↑: Increase or induction. If IC_50_ values were reported, we displayed them in the second column. If IC_50_ values were not reported, we only displayed effective concentrations in the second column.

**Table 13:** Antioxidant activity of SIN.

Activity	Cell Type or Model In Vitro (Effective Concentrations or IC_50_ Values)	Mechanism of Action	In Vivo		Year	Ref.
			Dose (mg/kg)	Therapeutic Effect		
Anti-oxidative stress	PC12 (5 μM)	↑: Nrf2 antioxidant system, cells survival rate, Nrf2, HO-1, NQO-1, and antioxidant genes expression. ↓: NOX and oxidative stress response.	NR		2017	[213]
Anti-GDM	NR	↓: IL-1β, TNF-α, IL-6, NF-κB, MYD88, NLRP3, TLR4, NF-κB, GDM, and TLR4.	10 mg/kg	↑: TAC, GST, and SOD. ↓: LPO. GP_X_,	2021	[214]
DPPH and H_2_O_2_ clearance	Mouse skin (DPPH: IC_50_ = 25.5 μM, H_2_O_2_: IC_50_ = 1.1 mM)	NR	5 mM	MDA inhibition	2022	[215]

NR: None reported. ↓: Decrease or inhibition. ↑: Increase or induction. If IC_50_ values were reported, we displayed them in the second column. If IC_50_ values were not reported, we only displayed effective concentrations in the second column.

**Table 14:** Drug–drug interaction.

Activity	Cell Type or Model In Vitro (Effective Concentrations or IC_50_ Values)	Mechanism of Action	In Vivo		Year	Ref.
			Dose (mg/kg)	Therapeutic Effect		
CYP2C19 inhibition	Cytochrome P450s (50 μM)	↑: Elimination of mephentoin promotion. ↓: CYP2C19.	NR		2007	[216]
Promoting drug absorption	Intestinal epithelial (0.5%, 1%, and 2% *w*/*v*)	↑: Apical-to-basolateral transport, drug absorption, tight junction transients, and molecular stability. ↓: TEER, basolateral-to-apical transport of the P-gp substrate cimetidine, ability of active efflux of P-gp substrates, and active drug efflux and transport.	NR		2010	[217]
Promoting intestinal absorption	Caco-2 cell (0.5% *w*/*v*)	↑: FD-4 flux, intestinal OCT absorption, and PKC signaling pathway. ↓: TEER and Claudin-1.	30 mg/kg	↑: Pharmacokinetic behavior of OCT. Intestinal absorption of OCT.	2013	[218]
Anti-RA	Jurkat T/PBMCs (0.03–3 μM)	↑: Jurkat T cells and normal PBMCs GR translocation regulatory. ↓: PBMCs proliferation and IC_50_ value of MP.	NR		2019	[219]

NR: None reported. ↓: Decrease or inhibition. ↑: Increase or induction. If IC_50_ values were reported, we displayed them in the second column. If IC_50_ values were not reported, we only displayed effective concentrations in the second column.

**Table 15:** Other bioactivities of SIN.

Activity	Cell Type or Model In Vitro (Effective Concentrations or IC_50_ Values)	Mechanism of Action	In Vivo		Year	Ref.
			Dose (mg/kg)	Therapeutic Effect		
Effects on teeth in rats	PDLSCs (0.1–2.0 M)	↑: ALP activity, deposition of mineralized nodules, OPG, ALP, and RUNX2. ↓: RANKL.	20, 40 mg/kg	↑: Alveolar bone structure, BV/TV, OPG, RUNX2, and OCN. ↓: Trap-positive osteoclasts on the compression side, RANKL, and TNF-α. OTM and root resorption inhibition.	2022	[220]
Promoting skin flap survival	HUVECs (80 μM)	↑: eNOS, autophagy flux, angiogenesis, cell apoptosis decrease, eNOS, flap survival, and PI3K/Akt pathway. ↓: Oxidative stress.	NR		2023	[221]
Improving benign prostatic hyperplasia	BPH-1 (25, 50 and 100 μM)	↑: Bax. ↓: SRD5A2, PCNA, BcL-2, and MMP2.	0.5, 1, 2 mg/kg	Decreased prostate gland (PG) weight coefficient in BPH mice	2023	[222]

NR: None reported. ↓: Decrease or inhibition. ↑: Increase or induction. If IC_50_ values were reported, we displayed them in the second column. If IC_50_ values were not reported, we only displayed effective concentrations in the second column.

## Data Availability

Not applicable.

## Abbreviations

BBB: blood–brain barrier; GI: gastrointestinal; P-gp: P-glycoprotein; A-THP-1: activated human monocyte THP-1; PGE2: prostaglandin E; COX-2: cyclooxygenase; CCK: cytokines cholecystokinin; MCP-1: monocyte chemotactic protein-1; ROS: reactive oxygen species; TFEB: transcription factor EB; GC: gastric cancer; NSCLC: non-small-cell lung cancer; HK2: hexokinases; MARCH1: membrane-associated ring finger protein; MFJD: Mufangji decoction; MPO: myeloperoxidase; HCC: hepatocellular carcinoma; TrxR: thioredoxin reductase; VM: vasculogenic mimicry; CAC: colitis-associated cancer; AOM: azoxymethane; DSS: dextran sulfate sodium; LPCAT3: lysophosphatidylcholine acyltransferase; FAO: fatty acid oxidation; ccRCC: clear-cell renal cell carcinoma; EMT: epithelial–mesenchymal transition; ER: endoplasmic reticulum; IR: ionizing radiation; ESCC: esophageal squamous cell carcinoma; NIS: sodium/iodide symporter; cAMP: cyclic adenosine monophosphate; RAI: radioactive iodine; RA: rheumatic arthritis; ACLA: aclarubicin; 5-FU: 5-fluorouracil; MDR: multidrug resistance; MTX: methotrexate; CIA: collagen-induced arthritis; OPG: osteoprotegerin; OPN: osteopontin; MM: multiple myeloma; GBM: glioblastoma multiform; SI: selective index; ESR: erythrocyte sedimentation rate; TNBS: trinitro-benzene-sulfonic acid; AGEs: advanced glycation end products; OMR: opioid μ-receptor; CHO: Chinese hamster ovarian; MSPGN: mesangial proliferative nephritis; GAG: glycosaminoglycan; FLS: fibroblast-like synovial cells; LMS: Liang Miao San; VCAM-1: vascular cell adhesion molecule; HMC-1: human hypertrophic cells; PMA: phorbol 12-myristate-13-acetate; RANKL: receptor activator of NF-κB ligand; ALP: intracellular alkaline phosphatase; Mt: M. tuberculosis; OCL: osteoclast-like cell; TNBS: 2,4,6-trinitrobenzenesulfonic acid; AIA: adjuvant induced Arthritis; CCI: chronic constriction injury; DRG: dorsal root ganglia; DNP: diabetic neuropathic pain; MWT: mechanical withdrawal threshold; TWL: thermal withdrawal latency; T2DM: type 2 diabetes mellitus; OGD/R: oxygen-glucose deprivation/reperfusion; MG: miroglia; VIP: vasoactive intestinal peptide; IP: inflammatory pain; CFA: complete Freund’s adjuvant; SOD: superoxide dismutase; MDA: malondialdehyde; CAT: catalase; OA: osteoarthritis; ECM: extracellular matrix; AS-TE: antioxidant surface transporter; CCAT1: colon-cancer-associated transcript-1; SIN-TSL: SIN thermosensitive liposome; LDH: lactate dehydrogenase; SNL: spinal nerve ligation; NP: neuropathic pain; RIP3: serine/threonine kinase 3; BMDMs: bone-marrow-derived macrophages; NLRP3: NOD-, LRR-, and pyrin domain-containing protein 3; GSTM1: glutathione S-transferase M1; LPS: lipopolysaccharide; ZO-1: zonula occluden-1; IA: indole-3-acrylic acid; IPA: indole-3-propionic acid; IAA: indole-3-acetic acid; AHR: aryl hydrocarbon receptor; PPARβ/δ: peroxisome-proliferator-activated receptor β/δ; BMDM: bone-marrow-derived macrophage; NETs: neutrophil extracellular trap; LC3-I: light chain 3-; LC3-II: LC3-phosphatidylethanolamine family protein conjugate; EAU: experimental autoimmune uveoretinitis; PD: Parkinson’s disease; Aβ: amyloid; TBI: traumatic brain injury; ARE: antioxidant response element; ICH: intracerebral hemorrhage; NREM: non-rapid eye movement; GAD: glutamic acid decarboxylase; CPP: conditioned place preference; PTZ: pentylenetetrazol; NMDAR: N-methyl-D-aspartic acid receptor; PTZ: pentylenetetrazol; MS: multiple sclerosis; MBP: myelin basic protein; GFAP: glial fibrillary acidic protein; MCAO: middle cerebral artery occlusion; TMT: trimethyltin; NOR: new object recognition; SAH: subarachnoid hemorrhage; EBI: early brain injury; SRBC: sheep red blood cell; PFC: plaque-forming cells; TECs: tubular epithelial cell; EAE: encephalomyelitis; DCs: dendritic cell; ZQFTN: Zhengqing FongtongNing; BDNF: brain-derived neurotrophic factor; pTrkB: p-neurotrophic receptor tyrosine kinas; pCREBP: phosphorylated camp response element-binding protein; CUMS: chronic unpredictable mild stress; 5-HT: 5-hydroxytryptamine; CLP: cecal ligation and puncture; BUN: urea nitrogen; Cr: creatinine; ALT: alanine transferase; AST: aspartate transaminase; PM: peritoneal macrophage; ALI: acute lung injury; CLP: cecal ligation and perforation; DOX: doxorubicin; p-IKK-β: phosphorylation of kidney lysate IKK-β; MPO: myeloperoxidase; UUO: unilateral ureteral obstruction; CAT: catalase; DN: diabetic nephropathy; HrGECs: human renal glomerular endothelial cell; HG: high glucose; TEER: trans-endothelial resistance; MCAO: middle cerebral artery occlusion; CRYAB: αB-crystallin; OGD: organic gastrointestinal disease; VSMC: vascular smooth muscle cell; PDGF: platelet-derived growth factor; HG: hyperglycemia; DM: diabetes mellitus; LVEDD: left-ventricle end-diastolic diameter; LVESD: left-ventricle end systolic diameter; IVSD: diastolic interventricular septal wall thickness; HR: heart rat; EF: ejection fraction; CH: cardiac hypertrophy; LVWI: left-ventricular weight index; LVAWd: the left-ventricular diastolic anterior wall thickness; LVPWd: the left-ventricular diastolic posterior wall thickness; VT: ventricular tachycardia; VF: ventricular fibrillation; VEB: ventricular ectopic beat; CK-MB: creatine kinase M; CK: creatine kinase; Tnl: troponin I; TXB 2: platelet aggregation parameter thromboxane B2; TF: tissue factor; Fbg: plasma fibrinogen; PA1-1: plasminogen activator inhibitor; GPx: glutathione peroxidase; TNF: tumor necrosis factor; APAP: alleviated acetaminophen; IR: ischemia reperfusion; OVA: ovalbumin; TRPV-l: transient receptor potential vanilloid-1; NKA: neurokinin A; GDM: gestational diabetes mellitus; LPO: lipid peroxidation; GST: glutathione S-transferase; TEER: transepithelial electrical resistance; OCT: octreotide; MP: methylprednisolone; GR: glucocorticoid receptor; PDLSCs: periodontal ligament stem cell; OTM: orthodontic tooth movement; BPH: benign prostatic hyperplasia; PG: prostate gland; TP: testosterone propionate; BMDC: bone-marrow-derived D; CTL: cytotoxic T lymphocyte; CsA: cyclosporine; CLP: cecal ligation puncture.
